# Sphingosine Kinase-1 Is Overexpressed and Correlates with Hypoxia in Osteosarcoma: Relationship with Clinicopathological Parameters

**DOI:** 10.3390/cancers14030499

**Published:** 2022-01-19

**Authors:** Anne Gomez-Brouchet, Claire Illac, Adeline Ledoux, Pierre-Yves Fortin, Sandra de Barros, Clémentine Vabre, Fabien Despas, Sophie Peries, Christelle Casaroli, Corinne Bouvier, Sébastien Aubert, Gonzague de Pinieux, Frédérique Larousserie, Louise Galmiche, Franck Talmont, Stuart Pitson, Marie-Lise Maddelein, Olivier Cuvillier

**Affiliations:** 1 CNRS, Institut de Pharmacologie et de Biologie Structurale, 31077 Toulouse, France; Illac.Claire@iuct-oncopole.fr (C.I.); ledouxadeline@yahoo.fr (A.L.); py.fortin85@gmail.com (P.-Y.F.); franck.talmont@ipbs.fr (F.T.); marie-lise.maddelein@ipbs.fr (M.-L.M.); 2 Université de Toulouse, UPS, 31400 Toulouse, France; 3 Département d’Anatomie et Cytologie Pathologies, Institut Universitaire du Cancer de Toulouse–Oncopôle (IUCT-O), 31059 Toulouse, France; 4 Cancer Biobank, Institut Universitaire du Cancer de Toulouse–Oncopôle (IUCT-O), 31059 Toulouse, France; Casaroli.Christelle@iuct-oncopole.fr; 5 Service de Pharmacologie Clinique, Hôpitaux de Toulouse, 31300 Toulouse, France; debarros.s@chu-toulouse.fr (S.d.B.); clementine.vabre@sfr.fr (C.V.); Despas.Fabien@iuct-oncopole.fr (F.D.); peries.s@chu-toulouse.fr (S.P.); 6 Department of Pathology, CHU la Timone, 13005 Marseille, France; Corinne.Bouvier2@ap-hm.fr; 7 Department of Pathology, CHRU de Lille, 50037 Lille, France; Sebastien.Aubert@chru-lille.fr; 8 Department of Pathology, CHU de Tours, 37044 Tours, France; gonzague.dubouexic@univ-tours.fr; 9 Department of Pathology, AP-HP, Hôpital Cochin, Universiteé Paris Descartes, 75014 Paris, France; frederique.larousserie@aphp.fr; 10 Centre Hospitalier Universitaire de Nantes Hôtel Dieu, 44000 Nantes, France; Louise.Galmiche@univ-nantes.fr; 11 Centre for Cancer Biology, University of South Australia and SA Pathology, Adelaide, SA 5000, Australia; Stuart.Pitson@unisa.edu.au

**Keywords:** FTY720, fingolimod, sphingosine kinase, S1P_1_, osteosarcoma, HIF, GLUT-1

## Abstract

**Simple Summary:**

Hypoxia has been recognized as a hallmark of solid tumors and a negative prognostic factor for response to therapeutics and survival of patients. Studies have demonstrated that the Sphingosine kinase-1/Sphingosine 1-Phosphate (SphK1/S1P) signaling pathway regulates the expression of the HIF-1 transcription factor in a number of solid tumor models, but no data are available in osteosarcoma characterized by hypoxia. The objectives of the present study were (i) to assess the contribution of SphK1/S1P signaling in regulating HIF-1α expression under hypoxia in various osteosarcoma cell models, (ii) quantify SphK1 enzymatic activity in biopsies of osteosarcoma, and (iii) examine the relationship between SphK1, S1P receptor 1 (S1P_1_) and hypoxia (GLUT-1) in 130 cases of osteosarcoma by immunohistochemistry. Our data suggest that the SphK1/S1P signaling might represent a potential target to investigate in osteosarcoma patients, considering that fingolimod, which inhibits SphK1 and the S1P_1_ receptor, is now reconsidered for repurposing in cancer.

**Abstract:**

The Sphingosine kinase-1/Sphingosine 1-Phosphate (SphK1/S1P) signaling pathway is overexpressed in various cancers, and is instrumental for the adaptation to hypoxia in a number of solid tumor models, but no data are available in osteosarcoma. Here we report that SphK1 and the S1P_1_ receptor are involved in HIF-1α accumulation in hypoxic osteosarcoma cells. FTY720 (Fingolimod), which targets SphK1 and S1P_1,_ prevented HIF-1α accumulation, and also inhibited cell proliferation in both normoxia and hypoxia unlike conventional chemotherapy. In human biopsies, a significant increase of SphK1 activity was observed in cancer compared with normal bones. In all sets of TMA samples (130 cases of osteosarcoma), immunohistochemical analysis showed the hypoxic marker GLUT-1, SphK1 and S1P_1_ were expressed in tumors. SphK1 correlated with the GLUT-1 suggesting that SphK1 is overexpressed and correlates with intratumoral hypoxia. No correlation was found between GLUT-1 or SphK1 and response to chemotherapy, but a statistical difference was found with increased S1P_1_ expression in patients with poor response in long bone osteosarcomas. Importantly, multivariate analyses showed that GLUT-1 was associated with an increased risk of death in flat bone, whereas SphK1 and S1P_1_ were associated with an increased risk of death in long bones.

## 1. Introduction

Although rare, osteosarcoma is the most common malignancy of bone predominantly affecting children and young adults [1,2]. It often occurs in the metaphyses of long bones such as the femur or tibia, and is pathologically characterized by cells with high-grade atypia and aberrant osteoid formation [1]. Jaw osteosarcoma accounts for only 6% of all osteosarcomas and develops mainly in the mandible. It is diagnosed approximately two decades later than long bone osteosarcoma and entails a lower risk of lung metastases reported to be 20–25% versus 44–49% for long bone osteosarcomas [3].

According to the 2020 WHO classification, osteosarcoma can be classified into high-grade conventional osteosarcomas, the most common histologic subtype (75% of all cases), periosteal osteosarcoma of intermediate-grade, and low-grade osteosarcomas (paraosteal and intramedullary osteosarcomas). Microscopically, conventional osteosarcoma is highly heterogeneous with cells that produce varying amounts of osteoid/chondroid matrix [2].

The high complexity of the osteosarcoma genome has not allowed for the identification of key molecular therapeutic targets so far [4,5,6]. As a consequence, osteosarcoma treatment still involves a combination of surgery with either neoadjuvant or adjuvant chemotherapy. The 5-year survival rate has not evolved over the past four decades, and does not exceed 70% with combined surgery and chemotherapy. It falls to 25% in patients with metastatic or relapsed osteosarcoma [7,8,9,10,11].

There are a number of reported mechanisms for treatment resistance in osteosarcoma including genomic aberrations, noncoding-RNA post-transcriptional regulations or tumor microenvironmental factors such as hypoxia [12]. Hypoxia is a feature of many solid human tumors as the aberrant cell proliferation rate is associated with a disequilibrium between oxygen supply and consumption [13]. The many effects of hypoxia on cancer biology include not only promotion of progression and metastasis [14] but also resistance to radiotherapy or chemotherapy [15]. The transcription factor, hypoxia-inducible factor 1α (HIF-1α), has been reported as the master driver of adaptation to hypoxia. A wealth of reports based on immunohistochemical studies of human tumor sections indicate that HIF-1α is overexpressed in the majority of human cancers and these elevated levels correlate with cancer-related death [16]. A number of studies have analyzed the association of hypoxic markers (HIF-1α, GLUT-1 or Glucose Transporter-1, and VEGF or Vascular Endothelial Growth Factor) with prognosis and clinicopathological characteristics of osteosarcoma. Recent meta-analyses suggest that hypoxia would be associated with lower survival rate, higher microvessel density, metastasis, higher pathologic grade, tumor stage and poor response to chemotherapy [17,18,19].

Sphingosine 1-phosphate (S1P) is a pleiotropic phospholipid that regulates proliferation, migration, inflammation or angiogenesis [20]. S1P is produced by phosphorylation of sphingosine by two isoforms of sphingosine kinases (SphK1 and SphK2), and irreversibly degraded into hexadecenal and ethanolamine phosphate by S1P lyase (SPL), an enzyme that has been shown to be downregulated in cancer [21]. Sphingosine kinase 1 (SphK1) is commonly overexpressed in cancer cells and correlates with poor patient prognosis in a number of tumor types [22,23]. As SphK1/S1P signaling contributes to malignant progression by controlling proliferation and metastatic potential of cancer cells, it represents a potential target for anticancer therapy [24]. We previously identified SphK1/S1P signaling as a new modulator of HIF-1α and HIF-2α activities under hypoxia in a wide array of cancer cell models (prostate, glioma, breast, lung and renal cell carcinoma) both in vitro and in vivo [25,26,27]. FTY720 or fingolimod (Figure S1) is an analogue of sphingosine that was approved by the US Food and Drug Administration (FDA) in 2010 for the treatment of relapsing–remitting multiple sclerosis after two phase III clinical studies establishing its efficacy and global safety [28,29]. It is currently under trial for the treatment of breast carcinoma, glioblastoma and anaplastic astrocytoma (NCT03941743 and NCT02490930). It can be phosphorylated in vivo to form FTY720-phosphate (FTY720-P), a mimetic of S1P, interacting with S1P receptors (excepted S1P_2_ subtype) but preferentially inducing internalization and degradation of S1P_1_, which has been associated with prevention of lymphocytes to egress from lymph nodes, reducing the amount of lymphocytes in peripheral blood [30]. Some of its actions are also attributed to its unphosphorylated form, notably inhibition of SphK1 activity associated with proteasomal degradation of SphK1, as suggested by several independent studies [31,32,33]. Antitumoral properties of FTY720 have been evidenced in a number of cancer animal models, including inhibition of angiogenesis and tumor vascularization [32,34,35,36,37]. In that respect, we previously reported that FTY720 decreases both HIF-1α and HIF-2α expression and subsequent expression of GLUT-1 and VEGF in a number of cellular and animal models of renal cell carcinoma [38]. Collectively, these data suggest that targeting of SphK1/S1P signaling represents a strategy that could potentially be exploited in therapeutic approaches to decrease hypoxia in cancer [39,40].

The objectives of this work were to assess (i) the role of the SphK1/S1P signaling in various cellular models (U-2OS, SaOS-2, MG-63 and 143B) of osteosarcoma cell lines under hypoxia, (ii) the SphK1 enzymatic activity in human osteosarcoma samples versus nontumoral bones, and (iii) the expression of HIF-1α target gene GLUT-1, SphK1 and S1P_1_ in 130 cases of osteosarcomas (long and flat bone samples) as the link between SphK1/S1P signaling and hypoxia has never been investigated in human tissues.

## 2. Materials and Methods

### 2.1. Patient and Tumor Characteristics

Tissue microarrays (TMAs) were prepared from the diagnostic biopsies of 130 patients coming from Toulouse (AGB), Lille (SA), Nantes (LG), Marseille (CB), Paris (FL), and Tours (GDP). TMAs (triplicate sampling of 1 mm) were established at the Institut Universitaire du Cancer biobank of Toulouse (AGB). For all cases, the TMA cores have been selected in the most cellular areas based on the diagnostic biopsy slide stained with hematoxylin–eosin (H&E). All OS samples were reviewed and reclassified by the accredited pathologists (SA, CB, FL, GDP, AGB) of the GFPO (French Group of Bone Pathologists), according to the WHO 2020 classification. The TMAs were then stored at the certified NF 96–900 cancer biobank of Toulouse (BB-0033-00014) where the immunohistochemistry study was conducted (GLUT1, SphK1, S1P_1_). In compliance with French law, the biobank cancer collection was declared to the Ministry of High Education and Research (DC-2020-4074) and a transfer agreement was obtained (AC-2020-4031) by ethical committees. All patient records and information were anonymized and deidentified before analysis. Informed consent was obtained from all patients and the use of the biological specimens was approved by the local institutional review board. Patient and tumor characteristics and response to treatment are described in Table 1 for the 130 patients included in the tissue microarray study. We studied 59 flat-bone osteosarcomas (mandibular and other locations: pelvis, ribs) and 71 long-bone osteosarcomas. To complete our exploration, we used 12 other surgical biopsies coming from the OS Toulouse collection and 10 normal bone samples also stored at the certified NF 96–900 cancer biobank of Toulouse (BB-0033-00014). These samples were used to quantify the SphK1 kinase enzymatic activity.

### 2.2. Immunohistochemistry

SphK1 immunohistochemistry was performed manually on deparaffinized FFPE tissue sections heat-pretreated (95°) using Envision TRS Low-pH buffer (pH 6, GV80511-2, Agilent Technologies, CA, USA) for 30 min. Sections were subsequently incubated with 3% hydrogen peroxide for 20 min to block endogenous peroxidase. The primary polyclonal SphK1 antibody [41], used at 1/300 (in Envision Flex diluent (K800621-2), Agilent Technologies), was applied for 1 h at room temperature and visualized using the Envision Flex DAB detection kit (Agilent Technologies). The tissue slides were counterstained using hematoxylin (Agilent Technologies) for 20 min at room temperature. S1P_1_ immunohistochemical stains were automated using the Discovery ULTRA (Roche, Ventana Medical Systems, Innovation Park Drive, Tucson, AZ, USA). After dewaxing, tissue slides were heat-pretreated using a CC1 (pH8) buffer (05424569001, Roche) for 32 min at 100 °C. The slides were blocked for endogenous peroxidase activity using the CM inhibitor (32 min at 37 °C) (Roche). The primary S1P_1_ antibody [42], used at 1/100 in Ventana Diluent (Roche) was incubated for 20 min at 36 °C. The target was then linked using the OmniMap anti-rabbit (05269679001, Roche, Tucson, AZ, USA) HRP-conjugated secondary antibody and visualized using the ChromoMap DAB detection kit (05266645001, Roche). The tissue slides were counterstained using hematoxylin (05277965001, Roche) for 8 min followed by postcoloration using the Bluing reagent for 4 min at room temperature (05266769001, Roche). Finally, GLUT1 immunohistochemistry was performed using the Benchmark ULTRA (Roche, Ventana Medical Systems, Tucson, AZ, USA). After dewaxing, tissue slides were heat-pretreated using a CC1 (pH 8) buffer (05424569001, Roche) for 64 min at 98 °C. The slides were blocked for endogenous peroxidase activity and incubated with ready-to-use primary anti-GLUT1 polyclonal antibody (06419178001, Roche) for 32 min. The target was then visualized using the OptiView DAB detection kit (06396500001, Roche). The tissue slides were counterstained using hematoxylin (05277965001, Roche) for 8 min followed by postcoloration using Bluing reagent for 4 min at room temperature (05266769001, Roche). All slides were then dehydrated (ethanol and xylene) and mounted using xylene-based mounting. Isotype negative-control immunoglobulin stains were included for all markers as quality control. The staining for each target was evaluated using two methods: the percentage of stained cells, without considering the intensity of the staining, altered by the preanalytical treatment (decalcification) of histological sections and the IRS Score (semiquantitative immunoreactive score). This latter scoring system multiplies staining intensity (4 grades) by the percentage of cells positive for the marker (5 grades) and results in a scoring of 0 to 12 [43,44]. Immunoreactivity was considered positive if detected in >1% of cells per core of 1mm, irrespective of staining intensity. A double-blind examination by two pathologists, one an expert in bone sarcoma, was performed for the interpretation of the immunohistochemistry results.

### 2.3. Chemicals and Reagents

Culture medium was obtained from Life Technologies (Saint Aubin, France). Serum was from Perbio (Brebières, France). [γ-32P]ATP was from Perkin-Elmer (Courtaboeuf, France). Silica gel 60 TLC plates were from VWR (Fontenay sous Bois, France). FTY720 (Fingolimod) was from Enzo Life Science (Villeurbanne, France). SKI II (CAS Number 312636-16-1) SphK1 inhibitor, doxorubicin and all other reagents were from Sigma-Aldrich (Saint-Quentin Fallavier, France).

### 2.4. Cell Lines

Human U-2OS, MG-63 and SaOS-2 osteosarcoma cell lines were obtained from ATCC (Molsheim, France). Human 143B cell line was kindly supplied by Dr F. Lecanda (CIMA, Pamplona, Spain). Cells were cultured in RPMI containing 10% fetal bovine serum at 37 °C in 5% CO_2_ humidified incubators. Cell lines were routinely verified by the following tests: morphology examination, growth analysis and mycoplasma detection (MycoAlert ^TM^, Lonza, Basel, Switzerland). All experiments were started with low-passaged cells (<25 times). Hypoxia (0.1% O_2_, 5% CO_2_, 94.5% N_2_) was achieved using an InVivo_2_ hypoxic workstation (Ruskinn, Bridgend, UK).

### 2.5. Sphingosine Kinase-1 Enzymatic Activity

SphK1 activity was performed as previously described [45], and determined in the presence of 50 µM sphingosine, 0.25% Triton X-100 and [γ-32P] ATP (10 µCi, 1 mM) containing 10 mM MgCl_2_. The labeled S1P was separated by thin-layer chromatography on silica gel 60 with 1-butanol/ethanol/acetic acid/water (80:20:10:10, *v/v*) and visualized by autoradiography. Activity was expressed as picomoles of S1P formed/min/mg of protein.

### 2.6. Western-Blot Analysis and Antibodies

Mouse anti-HIF-1α (BD Biosciences, Le Pont de Claix, France) was used as primary antibody. Proteins were visualized by an enhanced chemiluminescence detection system (GE Healthcare, Vélizy-Villacoublay, France) using anti-mouse horseradish peroxidase-conjugated IgG (Bio-Rad, Hercules). Equal loading of protein was confirmed by probing the blots with anti-α-tubulin or anti-ß-actin antibodies (Sigma-Aldrich). Densitometry quantitation was determined with Image J software 1.53i using the area under the peak method (NIH, Bethesda, MD, USA).

### 2.7. Quantitative Real Time PCR

Total RNA was isolated using the RNeasy minikit (Qiagen, Courtaboeuf, France) and 1 μg was reversed transcribed to cDNA using the SuperScript Frist Stand Synthesis System (Invitrogen). Quantitative real-time PCR was performed using the MESA Blue PCR Master mix (Eurogentec). Reactions were performed using hSphK1 (forward primer 5′-CTGGCAGCTTCCTTGAACCAT-3′; reverse primer, 5′-TGTGCAGAGACAGCAGGTTCA-3′), hSphK2 (forward primer 5′-CCAGTGTTGGAGAGCTGAAGGT-3′; reverse primer, 5′-GTCCATTCATCTGCTGGTCCTC-3′), hSPL (forward primer 5′-GAACCAAGTTGCAGTTCCCACCA-3′; reverse primer, 5′-ACAGTTGTCTGGGCCATGCCATAGA-3′), β-actin-specific primers (forward primer, 5′-GCAAAGACCTGTACGCCAAC-3′; reverse primer, 5′-AGT ACTTGCGCTCAGGAGGA-3′), TBP-specific primers (forward primer, 5′- TGACCTAAAGACCATTGCACTTCG-3′; reverse primer, 5′- CGTGGTTCGTGGCTCTCTTATC -3′). For analysis, all genes were normalized to expression of zeta polypeptide (YWHAZ) gene as endogenous control.

### 2.8. Cell Viability Assay

The MTT reagent (3-(4,5-dimethylthiazol-2-yl)-2,5-diphenyltetrazolium bromide) was used to determine cell death as previously described [46]. Briefly, cells were seeded at 5000 cells/well in 24-well plates and allowed to attach overnight. After 72 h of treatment, cells were incubated at 37 °C and 5% CO_2_ with 25 μL MTT solution (5 mg/mL; Sigma-Aldrich) for 4 h. After solubilization with 500 μL of lysis buffer (DMSO), formazan was quantified by spectrophotometry with a microplate reader at 570 nm absorbance. The GI_50_ values corresponding to the concentration that caused 50% inhibition of cell proliferation were calculated from dose–response curves obtained by nonlinear regression analysis. All the results were calculated from data obtained in three independent experiments.

### 2.9. RNA Interference Experiments

Transient interference was achieved by double-stranded human siRNAs 5′-GGGCAAGGCCUUGCAGCUCdTdT-3′ (siSphK1) as previously reported [25]. Aleatory sequence siScr was from Eurogentec (Angers, France). Transfections were carried out using Lipofectamine 2000 in OPTI-MEM medium according to the manufacturer’s instructions (Invitrogen, Villebon-sur-Yvette, France).

### 2.10. Statistical Analysis

The statistical significance of differences between the means of two groups was evaluated by unpaired Student’s *t* test. All statistical tests were two-sided, and the level of significance was set at *p* < 0.05. Calculations were performed using Prism 9 (GraphPad Software, San Diego, CA). For the overall survival analysis, the outcome was the occurrence of death, and the time was defined as the time between the date of diagnosis and the date of death (event) or the date of last follow-up (censorship). For the analysis of metastasis progression-free survival (MPFS), the outcome measure was the occurrence of metastasis and/or the occurrence of death. The time was defined as the time between the date of diagnosis and the date of metastatic progression, if there was presence of metastases, or the date of death (event) or the date of last follow-up (censorship). Patients who locally relapsed as their first event were considered to be censored data, in order to avoid the bias related to the quality of the surgical resection margins. All survival rates were estimated by the Kaplan–Meier method with 95% confidence intervals (CI). The association between the presence of markers expressed in percentage of cells (for a 10% increase in labeled cells) or using the IRS score (for an increase in the score of 1) and the occurrence of death or metastases/death was estimated using a Cox proportional hazards model with an adjustment for age. The association between the presence of markers and response to chemotherapy was estimated using logistic regression with adjustment for age. These multivariate analyzes were performed using SAS 9.4 software.

## 3. Results

### 3.1. The SphK1/S1P Signaling Regulates HIF-1α Accumulation in Osteosarcoma Cell Lines

**Figure 1:**
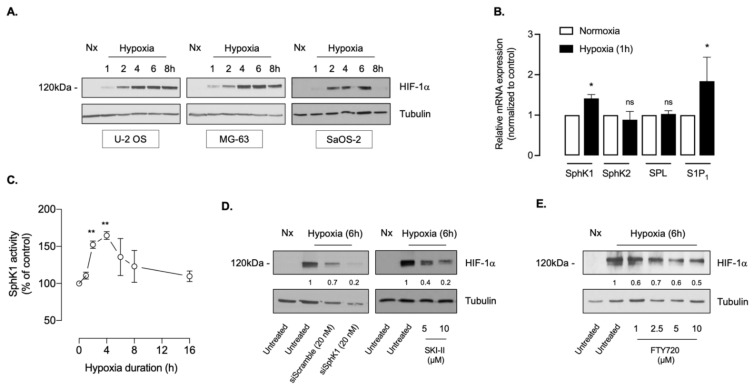
SphK1 inhibition by pharmacological inhibitors or RNA-silencing prevents HIF-1α accumulation in osteosarcoma. (**A**), human osteosarcoma U-2 OS, MG-63 and SaOS-2 cancer cells were incubated under normoxia or hypoxia up to 8 h. Cells were lysed and HIF-1α expression was analyzed by immunoblotting with an anti-HIF-1α antibody. Similar results were obtained in three independent experiments. (**B**), relative mRNA expression of SphK1, SphK2, S1P lyase (SPL) and S1P1 was measured in normoxic (Nx) and hypoxic U-2 OS cells. Columns; mean of at least three independent experiments, SEM. *, *p* < 0.05; n.s., not significant. (**C**), U-2 OS cells were incubated under hypoxia for the indicated times. Cell lysates were tested for SphK1 activity. Points; mean of five independent experiments, SEM. **, *p* < 0.01. Basal SphK1 activity was 290 ± 18 pmol/mg/min. (**D**), HIF-1α expression was analyzed on U-2 OS cells treated with the indicated concentrations of SKI-II (left) or with 20 nmol/L siSphK1 or scrambled siRNA. Similar results were obtained in three independent experiments. (**E**), U-2 OS cells were treated with the indicated concentrations of FTY720 and incubated under hypoxia for 6 h. Cell lysates were assayed for HIF-1α expression, including untreated normoxic cells (Nx). Similar results were obtained in three independent experiments. The uncropped Western blots have been shown in Figures S3–S5.

In agreement with work conducted by others [47,48,49,50,51,52], hypoxia was associated with increased HIF-1α accumulation in U-2 OS, MG-63 and SaOS-2 cell lines, peaking at 4–6 h of treatment (Figure 1A). Because we previously reported that the SphK1/S1P pathway is a regulator of both HIF-1α and HIF-2α during hypoxia in multiple cancer cell lineages [25,26,27,38], we evaluated its relevance with regard to HIF-1α expression in osteosarcoma cells. ln line with our previous findings in renal cell carcinoma models [27], we found an increase in SphK1 and S1P receptor 1 (S1P_1_) mRNA expression after 60 min of hypoxia in the U-2 OS cell model (Figure 1B). On the contrary, as shown in Figure 1B, there was no change of mRNA expression for both SphK2, the other sphingosine kinase isoform, and for S1P lyase (SPL), the S1P-degrading enzyme.

We next directly assessed SphK1 enzymatic activity. Hypoxia caused a rapid 2-fold increase in S1P production with a peak at 2–4 h, returning to basal levels by 16 h of treatment (Figure 1C). To validate that SphK1/S1P-dependent signaling was causal in the accumulation of HIF-1α, we first evaluated the effect of SphK1 knockdown with siRNA targeted against SphK1. Under hypoxia, SphK1 mRNA expression was markedly decreased in SphK1 RNAi-treated cells compared with those treated with scrambled RNAi (0.322 ± 0.04 versus 1.0, *n* = 3). In U2-OS cells, under hypoxia, siSphK1 treatment was associated with a significantly lower HIF-1α content (Figure 1D). We next used SKI-II, an inhibitor of SphK1 activity [53], and FTY720 that inhibits both SphK1 activity and S1P1 expression [30,31,32,33]. Both SKI-II and FTY720 dose-dependently inhibited HIF-1α accumulation in U-2 OS cells (Figure 1D,E). Collectively, these data suggesting that SphK1/S1P signaling is required for the regulation of HIF-1α in osteosarcoma are in line with our previous findings in prostate and renal cell carcinoma cell models [25,26,27,38]. Highly significant resistance to chemotherapies used in clinics including etoposide, cisplatin and doxorubicin have been reported in cellular models of osteosarcoma under hypoxic conditions [49,52]. In line with the aforementioned studies, hypoxia significantly reduced the sensitivity of U-2 OS, SaOS-2, MG-63 and 143B cells to doxorubicin with GI50 values exhibiting from a 3- to over 13-fold increase under hypoxia versus normoxic conditions (Table 1). In contrast, FTY720 remarkably reduced cell proliferation to the same extent in normoxic and hypoxic conditions in all osteosarcoma cell lines, suggesting that efficacy of FTY720 was related to its anti-HIF-1α effect.

### 3.2. Sphingosine Kinase-1 Activity Is Overexpressed in Osteosarcoma Biopsy Samples

**Figure 2:**
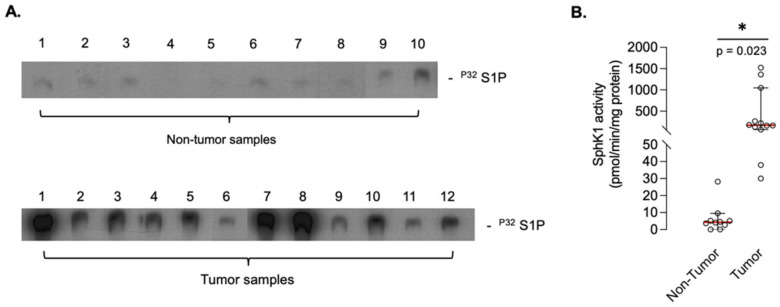
SphK1 activity in human osteosarcoma biopsy samples. (**A**), S1P content of 10 nontumoral and 12 tumoral biopsies as shown by thin-layer chromatography. (**B**), SphK1 enzymatic activity of biopsy samples expressed as pmol/min/mg of protein. Columns are individual SphK1 activity values and means with 95% CI. Mean SphK1 activities were 6.3 (95% CI: 0.45–12.1) and 432 (95% CI: 77.4–835) pmol/mg/min for nontumoral and tumoral biopsy samples, respectively. *, *p* < 0.01. The uncropped Thin-Layer Chromatography films have been shown in Figure S6.

Sphingosine kinase-1 (SphK1) enzymatic activity was assessed in human biopsies (10 nontumor and 12 tumor individual samples). As a whole, mean SphK1 activity was 432 pmol/min/mg (95% CI: 77.4–835) in tumor samples versus 6.3 (95% CI: 0.45–12.1) in noncancerous tissue, accounting for a statistically significant (*p* = 0.023) increase in osteosarcoma tissue compared to normal bone, suggesting that S1P is abundantly generated in osteosarcoma tissues (Figure 2).

### 3.3. Description of the Patient Population

The demographic, clinical and histological data of the 130 patients are summarized in Table 2. The median age at diagnosis was 22 years (range, 6 to 84). Further, 61 of the patients (56.9%) were >25 years of age and 80 (61.5%) were males. Additionally, 59 osteosarcomas were located in the flat bones (mandibular and other locations) (45.4%) and 71 in the limbs (54.6%). All osteosarcomas were high-grade, conventional subtypes with a higher proportion conventional osteoblastic (46.2%) compared to chondroblastic (24.6%). The tumors were localized in 89 (68.5%) of cases and there were 41 (31.5%) of metastatic patients.

We compared the clinical parameters between the two distinct groups: flat- versus long-bone osteosarcomas. The latter were significantly more common in young people under the age of 25 (*p* < 0.001) and in men (*p* = 0.0068). Osteosarcomas localized to the mandible/flat bone were mainly seen in older patients (*p* < 0.001). Metastases were significantly more common in patients with long-bone osteosarcomas (*p* = 0.012). The response to chemotherapy was significantly better in patients with long-bone osteosarcomas compared to other locations (*p* = 0.0064).

### 3.4. Immunohistochemistry Analysis of GLUT-1, SphK1 and S1P_1_ Expression

**Figure 3:**
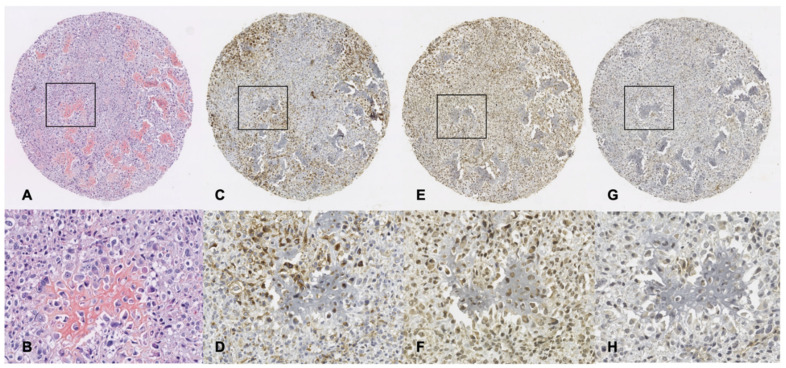
Representative patterns of GLUT-1, SphK1 and S1P_1_ expression in osteosarcoma. (**A**), Histological representation of one spot of an osteoblastic osteosarcoma included in TMAs (H&E magnification ×5), (**B**), Representation at a magnification ×25 of the zone framed in A; (**C**,**D**), GLUT-1 staining in the same spot (magnification ×5 and 25, respectively); (**E**,**F**), SphK1 staining in the same spot (magnification ×5 and 25, respectively); (**G**,**H**), S1P_1_ staining in the same spot (magnification ×5 and 25, respectively).

GLUT-1 expression was found in 49.3% of all patients (*n* = 98) with an IRS score of 6.31 (Table 3). The staining pattern of GLUT-1 was mostly membranous, with some staining or light dusting in the cytoplasm, as is characteristic of GLUT-1 immunohistochemistry (Figure 3C,D). A significantly higher percentage of cells stained for GLUT-1 and a higher IRS score were associated with long-bone (*p* = 0.0116 and *p* = 0.0353, respectively) versus flat-bone osteosarcomas (Table 3). SphK1 and S1P_1_ expressions were found in 67.3% of all patients (*n* = 96) with an IRS score of 4.547, and in 64.2% of all patients (*n* = 111) with an IRS score of 4.3, respectively (Table 3). Of note, there were no statistical differences of SphK1 and S1P_1_ (percentage of stained cells or IRS score) between the osteosarcoma sites (Table 3). The staining pattern of SphK1 (Figure 3E,F) was cytoplasmic as we previously reported in prostate and lung cancer [21,22,23]. The staining pattern of S1P_1_ (Figure 3G,H) was membranous and cytoplasmic as we previously reported in kidney cancer [42]. As shown in Table 4, although the strength of linear correlation between SphK1 and GLUT-1 was weak, it was statistically extremely significant for all osteosarcomas (*p* = 0.005 for percentage of stained cells and *p* = 0.001 for IRS score). Similar statistical correlation, albeit to a lesser extent, was found for the long-bone (percentage of stained cells and IRS score) and the flat-bone osteosarcoma groups (IRS score only). On the contrary, no statistical correlations were found between SphK1 and S1P_1_ or between GLUT-1 and S1P_1_.

### 3.5. Clinical Parameters and Biomarkers Association with Treatment Response

To study the association with response to chemotherapy, we evaluated the correlation between the extent of response to treatment assessed by the histological grading scale of Salzer-Kuntschik et al. [54] and clinical parameter or each biomarker (GLUT-1, SphK1 or S1P_1_). A good response to chemotherapy was defined as <10% viable tumor cells in the surgical specimen, while a poor response was defined as ≥10% viable tumor cells in the surgical specimen. For all osteosarcomas, patients with a good response were younger (*p* < 0.0001) (Table S1) and there was no association between the response to treatment and the expression of GLUT-1, SphK1 or S1P_1_ (Table 5). Regarding long-bone osteosarcomas, statistical differences were found with an increased expression of S1P_1_ in poor-responder patients (Table 5).

### 3.6. Clinical Parameters and Biomarkers Associated with Survival in the Different Subgroups (Long Bones Versus Flat Bones and Younger Versus Older Patients)

Survival analyses (Figure S2) showed no statistically significant difference in overall survival and metastasis-free survival between long- and flat-bone osteosarcoma patients (*p* = 0.3307 and *p* = 0.468, respectively). As shown in Table S2, univariate analysis showed that S1P_1_ expression (% of stained cells) was statistically associated with an increased risk of death HR = 1.31 (95% CI: 1.03–1.67), or metastatic progression HR 1.22 (95% CI: 1.02–1.45), and poorer response to chemotherapy HR 0.71 (95% CI: 0.51–0.98) in long bones.

Age-adjusted multivariate analyses are presented in Table 6. Regarding overall survival for the flat-bone osteosarcomas, the expression of GLUT-1 (% of stained cells and IRS score) was associated with an increased risk of death HR = 1.20 (95% CI: 1.00–1.44) and HR = 1.15 (95% CI: 1.00–1.32), respectively. With regard to long bones, the expression of GLUT-1 (% of stained cells and IRS score) was, associated with an increased risk of death HR = 1.16 (95% CI: 0.98–1.38) and HR = 1.14 (95% CI: 1.00–1.29) with borderline significance (0.08 and 0.06, respectively). SphK1 expression was associated with a significant increase in the risk of death HR = 1.25 (95% CI: 1.03–1.51) only in the long-bone patients (based on IRS score). Similarly, in long-bone osteosarcomas, S1P_1_ expression was associated with an increased risk of death HR = 1.29 (95% CI: 1.00–1.66) at the limit of significance (based on % of stained cells) with regard to overall survival. S1P_1_ expression was associated with a significant increase in the risk of metastasis in the long-bone osteosarcomas group, HR = 1.23 (95% CI: 1.02–1.48). No statistical difference was found for S1P_1_ expression expressed as IRS score. Finally, no significant differences were found in the good responders to chemotherapy according to the expression of markers expressed as % of stained cells or IRS score for all markers: GLUT1, SphK1 and S1P_1_.

## 4. Discussion

Hypoxia is a characteristic of solid tumors, and the adaptation of cancer cells to hypoxia is instrumental in the development of aggressive phenotype and associated with a poor prognostic in patients [55]. At the cellular level, the adaptation to hypoxia is predominantly mediated by the hypoxia-inducible factors (HIFs), consisting of an oxygen-sensitive α-subunit and a constitutively expressed β-subunit, that regulate the expression of target genes promoting angiogenesis such as VEGF, glycolysis such as GLUT-1, acidosis such as CA-IX (carbonic anhydrase IX), metastasis, increased tumor growth and resistance to treatments [16].

Immunohistochemical-based analyses (HIF-1α, GLUT-1, VEGF, CA-IX…) have shown a connection between hypoxia and outcome of tumor therapy. In osteosarcoma, initial studies examined VEGF expression, which was found to be associated with poor outcome (DFS, OS) and metastasis [56,57,58]. As a target gene of HIF transcription factors, VEGF was also correlated with HIF-1α expression [57,58]. Most studies have examined the expression of HIF-1α suggesting that it would be associated with higher pathologic grade and tumor stage, higher MVD (microvascular density), higher rate of metastasis, poorer overall survival, poorer disease-free survival and poor response to chemotherapy [48,50,51,58,59,60,61,62,63]. Three meta-analyses recently concluded that HIF-1α expression might be an effective predictive factor of poor prognosis in osteosarcoma [17,18,19]. As pointed out by the authors, the results of these meta-analyses should be interpreted with caution as some major biases could not be excluded such as the limited sample size of nearly all studies, the fact that most of the selected studies have been carried out in China, proposing that similar studies have to be conducted in populations of other origins, the heterogeneity across the assessment of HIF-1α expression (different antibodies or dilutions that could affect the sensitivity) as well as nonuniform criteria including different cut-off values defining high HIF-1α level of expression. Fewer studies have examined GLUT-1 expression in canine and human osteosarcomas leading to conflicting results [64,65,66] ranging from no statistical correlation between GLUT-1 expression and DFS, survival time or percentage of necrosis in dogs [64], to strong GLUT-1 staining in both primary sites and metastases [65] and association with shorter DFS [66]. In addition, these studies of GLUT-1 expression are also limited by the size of the samples (*n* = 10 to 44). More recently, a study examining the expression of plasma GLUT-1 established that GLUT-1 was overexpressed in patients with osteosarcoma (*n* = 42) compared to healthy volunteers (*n* = 38). In addition, levels of GLUT-1 mRNA were significantly higher in tumor tissues than in adjacent healthy tissues of osteosarcoma patients [67].

In this work, we have used TMAs from different origins, which is therefore a material, obtained with different fixation and decalcification conditions, that does not allow for reliable evaluation of HIF-1α expression, with an antibody known to be difficult to use in routine. For this reason, we relied on GLUT-1 staining using a more robust antibody used in clinical practice. We found that GLUT-1 was expressed in both long and flat bones with a higher expression of GLUT-1 in long bones. Although no correlation was found with response to chemotherapy, GLUT-1 expression was associated with a higher risk of death (OS) in flat bones and a tendency for death or metastatic progression (MPFS). These data thus suggest that hypoxia might indeed be an effective predictive factor of poor prognosis in osteosarcoma, in line with the previously reported studies with HIF-1α [48,50,51,58,59,60,61,62,63], the transcription factor that regulates GLUT-1 under hypoxia. We also examined the relationship between hypoxia (GLUT-1) and the SphK1/S1P signaling, which has been previously documented by our team to be central for the adaptation to hypoxia in a wide array of cellular (prostate, glioblastoma, lung, breast, renal cell carcinoma) and animal (prostate, renal cell carcinoma) models [25,26,27,38].

SphK1/S1P signaling is commonly upregulated in cancer cells [24] and correlates with poor patient prognosis in a number of tumor types (reviewed in [68]). The expression of SphK1 in osteosarcoma had never been reported until this work. A recent study found that S1P levels in serum samples from osteosarcoma patients (*n* = 17) are decreased after chemotherapy [69], suggesting that S1P could represent a potential biomarker of chemotherapeutic efficacy. As the authors did not assess serum S1P levels in healthy controls, they could not demonstrate that S1P content is in fact increased in osteosarcoma patients.

In our tumor collection, both SphK1 and S1P_1_ were expressed in long and flat bones together with GLUT-1. A strong correlation was essentially seen between GLUT-1 and SphK1, regardless of the location (flat versus long bones), suggesting that SphK1 activation is associated with hypoxia in vivo. No association between the response to treatment and the expression of GLUT-1, SphK1 or S1P_1_ was found, with the exception of long-bone osteosarcomas where statistical differences were found with an increased expression of S1P_1_ in poor-responder patients. These data regarding response to chemotherapy should be interpreted with caution, as the different groups of patients (long- versus flat-bone osteosarcomas) are not always treated with the same chemotherapy regimen. Importantly, age-adjusted multivariate analyses showed that GLUT-1, SphK1 and S1P_1_ were associated with an increased risk of death (OS) depending on the osteosarcoma location (long versus flat bones). Interestingly, S1P_1_ was the only biomarker associated with increased risk of death (OS), increased risk of death or metastasis (MPFS) and predictive of poorer response to chemotherapy in long bones. However, one should be cautious in interpreting these findings on bone tissues as the staining intensity of S1P_1_ was weaker when compared to the robust GLUT-1 and SphK1 antibodies. The strong expression of SphK1 in tissues was confirmed by the quantification of SphK1 enzymatic activity, which we originally reported in prostate cancer patients [22], using 12 osteosarcoma compared to 10 nontumoral bone samples. We found that SphK1 activity was dramatically augmented in osteosarcoma with over a 50-fold increase in S1P production (431 pmoles/min/mg versus 6.6, *p* = 0.023). Measuring enzymatic activity in human biopsies is technically demanding and hardly amenable to routine use as a prognostic factor, yet it provides the most accurate information by directly quantifying the activity of an enzyme when compared to mRNA or protein assessment. The relationship between SphK1/S1P signaling and hypoxia was further validated in vitro in various osteosarcoma cell lines exposed to hypoxic conditions. Our data suggesting that SphK1/S1P signaling activation is associated with HIF-1α expression in osteosarcoma are in line with our previous findings in prostate and renal cell carcinoma cell models [25,26,27,38]. Interestingly, in contrast to classical chemotherapy, the dual SphK1 and S1P_1_ inhibitor FTY720 remarkably reduced cell proliferation to the same extent in normoxic and hypoxic conditions in all osteosarcoma cell lines, suggesting that efficacy of FTY720 was related to its anti-HIF-1α effect.

## 5. Conclusions

Our data (in vitro, enzymatic assessment of SphK1 activity in tumors and SphK1 and/or S1P1 expression in tissues) suggest that SphK1/S1P signaling might represent a potential target to investigate in osteosarcoma patients, considering that the therapeutic drug FTY720 (also known as fingolimod, and marketed by Novartis as Gilenya^TM^) is already used in clinic for multiple sclerosis and is now being considered for repurposing in cancer.

## Figures and Tables

**Table 1:** Patient and tumor characteristics.

Variable	All		Long Bones		Flat Bones		*p* Value
	*n*	%	*n*	%	*n*	%	
	130	100	71	54.6	59	45.4	
Age (Years)							
Median (Range)	22.4 (5.7–83.9)		15.8 (6.6–82.8)		39 (5.7–83.9)		<0.0001
<25 (Years)	69	53.1	53	40.8	16	12.3	<0.0001
≥25 (Years)	61	46.9	18	13.8	43	33.1	
Gender							0.0068
Male	80	61.5	51	39.2	29	22.3	
Female	49	37.7	20	15.4	29	22.3	
Unknown	1	0.8	0	0.0	1	0.8	
Site							
Flat bones	59	45.4					
Long bones	71	54.6					
Histological type							0.8099
Chondroblastic	32	24.6	11	8.5	21	16.2	
Osteoblastic	60	46.2	47	36.2	13	10.0	
Fibroblastic	25	19.2	7	5.4	18	13.8	
Other	10	7.7	4	3.1	6	4.6	
Unknown	3	2.3	2	1.5	1	0.8	
Metastatic vs Non Metastatic							0.012
Localized	89	68.5	42	32.3	47	36.2	
Metastatic	41	31.5	29	22.3	12	9.2	
Response to chemotherapy							0.0064
Good	53	40.8	39	30.0	14	10.8	
Poor	43	33.1	20	15.4	23	17.7	
Unknown	34	26.2	12	9.2	22	16.9	

**Table 2:** Cytotoxicity of doxorubicin and FTY-720 on a representative panel of osteosarcoma cell lineages (GI_50_, 72 h, MTT assay).

Variable	Doxorubicin		*FOLD*	FTY720		*FOLD*
	Normoxia	Hypoxia		Normoxia	Hypoxia	
U-2 OS	0.066	0.195	3	4.21	4.21	1
SaOS-2	0.073	1.00	14	3.20	3.23	1
MG-63	0.025	0.507	20	1.52	1.50	1
143B	0.017	0.134	8	1.11	1.22	1

The GI_50_ values represent the concentration of compound causing 50% inhibition of cell growth. Mean of at least three independent experiments.

**Table 3:** Immunohistochemical results for GLUT-1, SphK1 and S1P_1_.

Variable	All					Long Bones					Flat Bones					*p* Value
	*AVG*	SD	Median	Min	Max	*AVG*	SD	Median	Min	Max	*AVG*	SD	Median	Min	Max	
GLUT1																
*n* = 98						*n* = 54					*n* = 44					
% of stained cells	49.29	31.73	60	0	100	55.37	32.95	65	0	100	41.82	28.8	50	0	90	0.0116
IRS score	6.31	4.03	6	0	12	7.06	4.03	9	0	12	5.39	3.88	6	0	12	0.0353
SphK1																
*n* = 96						*n* = 52					*n* = 44					
% of stained cells	67.28	28.29	80	0	100	70.77	26.7	80	0	100	63.16	29.83	80	0	90	0.1835
IRS score	4.57	2.7	4	0	12	4.85	2.55	6	0	12	4.25	2.86	3	0	12	0.1828
S1P_1_																
*n* = 111						n = 63					*n* = 48					
% of stained cells	64.23	23.14	70	0	100	64.29	23.6	70	0	100	64.17	22.77	70	0	90	0.9279
IRS score	4.3	2.52	3	0	12	4.25	2.67	3	0	12	4.35	2.33	4	0	12	0.6881

**Table 4:** Correlation between GLUT-1, SphK1 and S1P_1_ markers.

**All OS**							
correlation coefficient (r)	GLUT1 % cells	SphK1 % cells	S1P_1_ % cells	correlation coefficient (r)	GLUT1 IRS score	SphK1 IRS score	S1P_1_ IRS score
GLUT1 % cells	NA	0.3019	0.009351	GLUT1 IRS score	NA	0.3498	−0.01708
SphK1 % cells		NA	0.1387	SphK1 IRS score		NA	−0.01275
S1P_1_ % cells			NA	S1P_1_ IRS score			NA
*p* value	GLUT1 % cells	SphK1 % cells	S1P_1_ % cells	*p* value	GLUT1 IRS score	SphK1 IRS score	S1P_1_ IRS score
GLUT1 % cells	NA	0.005	0.9287	GLUT1 IRS score	NA	0.001	0.8702
SphK1 % cells		NA	0.1922	SphK1 IRS score		NA	0.9051
S1P_1_ % cells			NA	S1P_1_ IRS score			NA
**Long Bones**							
correlation coefficient (r)	GLUT1 % cells	SphK1 % cells	S1P_1_ % cells	correlation coefficient (r)	GLUT1 IRS score	SphK1 IRS score	S1P_1_ IRS score
GLUT1 % cells	NA	0.3608	−0.1556	GLUT1 IRS score	NA	0.2902	−0.1024
SphK1 % cells		NA	0.1765	SphK1 IRS score		NA	−0.1345
S1P_1_ % cells			NA	S1P_1_ IRS score			NA
*p* value	GLUT1 % cells	SphK1 % cells	S1P_1_ % cells	*p* value	GLUT1 IRS score	SphK1 IRS score	S1P_1_ IRS score
GLUT1 % cells	NA	0.0127	0.2706	GLUT1 IRS score	NA	0.0478	0.4699
SphK1 % cells		NA	0.2153	SphK1 IRS score		NA	0.3466
S1P_1_ % cells			NA	S1P_1_ IRS score			NA
**Flat Bones**							
correlation coefficient (r)	GLUT1 % cells	SphK1 % cells	S1P_1_ %cells	correlation coefficient (r)	GLUT1 IRS score	SphK1 IRS score	S1P_1_ IRS score
GLUT1 % cells	NA	0.1979	0.174	GLUT1 IRS score	NA	0.3948	0.09562
SphK1 % cells		NA	0.1661	SphK1 IRS score		NA	0.2053
S1P_1_ % cells			NA	S1P_1_ IRS score			NA
*p* value	GLUT1 % cells	SphK1 % cells	S1P_1_ %cells	*p* value	GLUT1 IRS score	SphK1 IRS score	S1P_1_ IRS score
GLUT1 % cells	NA	0.2336	0.2703	GLUT1 IRS score	NA	0.0142	0.5469
SphK1 % cells		NA	0.3058	SphK1 IRS score		NA	0.2039
S1P_1_ % cells			NA	S1P_1_ IRS score			NA

**Table 5:** GLUT-1, SphK1 and S1P_1_ expression of good and poor responders to chemotherapy.

All OS	Good					Poor					*p* Value
	*n* = 40					*n* = 30					
GLUT1	AVG	SD	Median	Min	Max	AVG	SD	Median	Min	Max	
% of stained cells	57.75	31.73	70	0	100	45	29.8	50	0	90	0.1945
IRS score	7.4	3.85	9	0	12	5.67	3.94	6	0	12	0.0587
SphK1	*n* = 38					*n* = 31					
% of stained cells	69.74	25.09	80	0	100	68.39	25.96	80	0	90	0.9506
IRS score	4.76	2.8	5	0	12	4.45	2.39	4	0	8	0.7955
S1P_1_	*n* = 45					*n* = 36					
% of stained cells	57.78	23.54	60	0	90	66.11	24.06	70	0	90	0.2325
IRS score	3.69	2.2	3	0	12	4.58	2.71	3	0	12	0.0787
Long Bones	*n* = 39					*n* = 20					*p* value
GLUT1	AVG	SD	Median	Min	Max	AVG	SD	Median	Min	Max	
% of stained cells	58.97	31.89	70	0	100	56.67	30.86	60	0	90	0.8059
IRS score	7.59	3.78	9	0	12	7	4.05	9	0	12	0.6482
SphK1	*n* = 28					*n* = 13					
% of stained cells	75	17.53	80	0	100	69.23	29.85	80	0	90	0.744
IRS score	5	2.33	6	0	12	4.54	2.37	4	0	8	0.6947
S1P_1_	*n* = 34					*n* = 18					
% of stained cells	56.18	23.1	60	0	90	71.67	23.33	80	0	90	0.0038
IRS score	3.47	2.22	3	0	12	5.22	2.94	6	0	12	0.0098
Flat Bones	*n* = 11					*n* = 15					*p* value
GLUT1	AVG	SD	Median	Min	Max	AVG	SD	Median	Min	Max	
% of stained cells	54.55	32.67	60	0	90	33.33	24.4	40	0	70	0.0569
IRS score	6.91	4.18	9	0	12	4.33	3.44	6	0	9	0.0993
SphK1	*n* = 10					*n* = 18					
% of stained cells	55	36.59	70	0	90	67.78	23.65	75	0	90	0.5847
IRS score	4.1	3.93	3	0	12	4.39	2.48	3	0	8	0.4539
S1P_1_	*n* = 11					*n* = 18					
% of stained cells	62.73	25.33	80	20	90	60.56	24.13	65	0	90	0.7043
IRS score	4.36	2.11	4	2	8	3.94	2.36	3	0	9	0.6521

**Table 6:** Age-adjusted multivariate analyses.

Variable	Death (Overall Survival)			Death or Metastatic Progression (MPFS)			Good Response to Chemotherapy		
	All OS	Long Bones	Flat Bones	All OS	Long Bones	Flat Bones	All OS	Long Bones	Flat Bones
GLUT1 (% of cells) *									
*n*	98	54	44	98	54	44	70	44	26
HRa	1.03	0.92	1.2	1.05	0.97	1.16	1.09	0.92	1.32
[IC 95%] **	[0.93–1.15]	[0.80–1.06]	[1.00–1.44]	[0.96–1.15]	[0.87–1.08]	[0.98–1.38]	[0.92–1.29]	[0.72–1.19]	[0.95–1.84]
*p*	0.57	0.24	0.05	0.31	0.58	0.08	0.34	0.53	0.1
SphK1 (% of cells) *									
*n*	96	52	44	96	52	44	69	41	28
HRa [IC 95%] **	1.04	1.1	0.9	1.03	1.06	0.95	0.98	1.04	0.82
	[0.88–1.15]	[0.90–1.36]	[0.74–1.11]	[0.91–1.16]	[0.90–1.25]	[0.79–1.16]	[0.79–1.21]	[0.73–1.47]	[0.62–1.10]
*p*	0.96	0.36	0.33	0.68	0.46	0.63	0.82	0.83	0.19
S1P_1_ (% of cells) *									
*n*	111	63	48	111	63	48	81	52	29
HRa [IC 95%] **	1.09	1.29	0.92	1.01	1.23	0.91	0.88	0.72	1.07
	[0.94–1.27]	[1.00–1.66]	[0.75–1.13]	[0.96–1.26]	[1.02–1.48]	[0.74–1.11]	[0.71–1.09]	[0.52–1.01]	[0.6–1.51]
*p*	0.27	0.05	0.42	0.17	0.03	0.35	0.24	0.06	0.7
GLUT1 (IRS Score) *									
*n*	98	54	44	98	54	44	70	44	26
HRa [IC 95%] **	1.05	0.97	1.15	1.05	0.99	1.14	1.09	0.98	1.2
	[0.96–1.14]	[0.86–1.09]	[1.00–1.32]	[0.98–1.13]	[0.90–1.08]	[1.00–1.29]	[0.95–1.25]	[0.80–1.19]	[0.94–1.54]
*p*	0.27	0.58	0.04	0.17	0.87	0.06	0.23	0.82	0.13
SphK1 (IRS Score) *									
*n*	96	52	44	96	52	44	69	41	28
HRa [IC 95%] **	1.07	1.25	0.9	1.03	1.13	0.94	0.95	0.94	0.93
	[0.93–1.23]	[1.03–1.51]	[0.71–1.14]	[0.92–1.16]	[0.96–1.35]	[0.77–1.16]	[0.77–1.17]	[0.66–1.34]	[0.71–1.23]
*p*	0.35	0.02	0.39	0.6	0.15	0.58	0.63	0.73	0.62
S1P_1_ (IRS Score) *									
*n*	111	63	48	111	63	48	81	52	29
HRa [IC 95%] **	1.05	1.07	1.02	1.07	1.09	1.01	0.89	0.8	1.1
	[0.94–1.18]	[0.92–1.23]	[0.84–1.24]	[0.97–1.18]	[0.97–1.21]	[0.83–1.24]	[0.73–1.08]	[0.62–1.03]	[0.78–1.55]
*p*	0.4	0.39	0.86	0.18	0.14	0.89	0.22	0.08	0.6

* for a 10% increase of stained cells; ** Age-adjusted.

## Data Availability

Data are contained within the article or Supplementary Materials.

## Supplementary Materials

The following are available online at https://www.mdpi.com/article/10.3390/cancers14030499/s1, Figure S1: Structure of FTY720, Figure S2: Kaplan–Meier survival curves for overall survival and metastasis progression-free survival according to osteosarcoma location, Figure S3: Original film (Figure 1A), Figure S4: Original films (Figure 1E), Figure S5: Original films. Figure 1D, Figure S6: Original radiography obtained from Thin Layer Chromatography for experiment described in Figure 2A, Table S1: Clinical parameters of good and poor responders to chemotherapy, Table S2: Association between GLUT1, SphK1 and S1P_1_ biomarkers and OS, MPFS and good response to chemotherapy. Comparison between long-bone and flat-bone locations.
