# Supplementary material for: Sphingosine Kinase-1 Is Overexpressed and Correlates with Hypoxia in Osteosarcoma: Relationship with Clinicopathological Parameters
Source: Cancers (Basel). 2022 Jan 19;14(3):499. doi: 10.3390/cancers14030499 (PMC8833796; doi:10.3390/cancers14030499)
Supplement: Supplementary file 1 [file cancers-14-00499-s001.zip › cancers-1523534-supplementary.pdf]

cancers

Supplementary Materials: Sphingosine Kinase-1 is Overexpressed and Correlates with Hypoxia in Osteosarcoma: Relationship with Clinicopathological Parameters

Anne Gomez-Brouchet, Claire Illac, Adeline Ledoux, Pierre-Yves Fortin, Sandra de Barros, Clémentine Vabre, Fabien Despas, Sophie Peries, Christelle Casaroli, Corinne Bouvier, Sébastien Aubert, Gonzague de Pinieux, Frédérique Larousserie, Louise Galmiche, Franck Talmont, Stuart Pitson, Marie-Lise Maddelein and Olivier Cuvillier

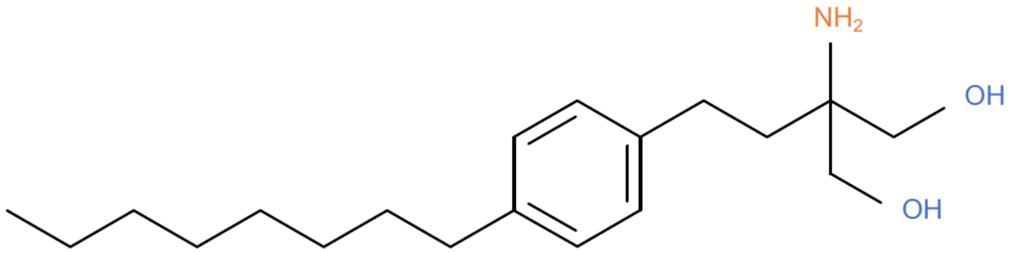

Figure S1. Structure of FTY720.

A.

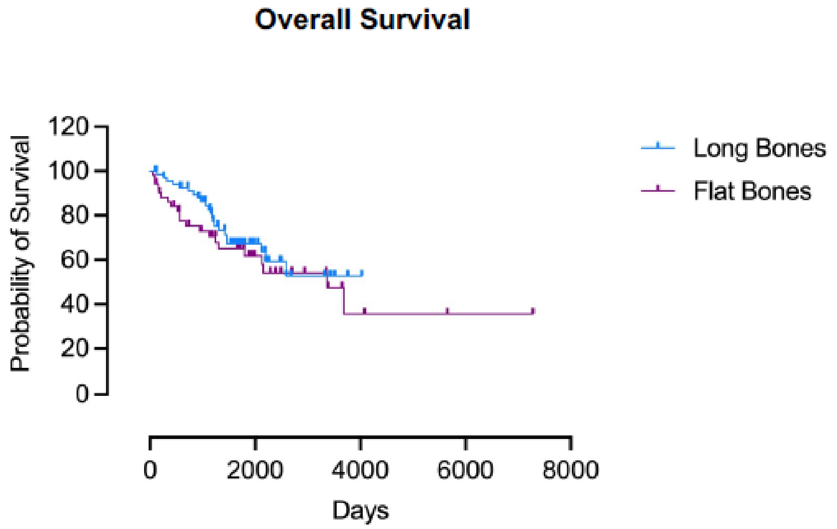

B.

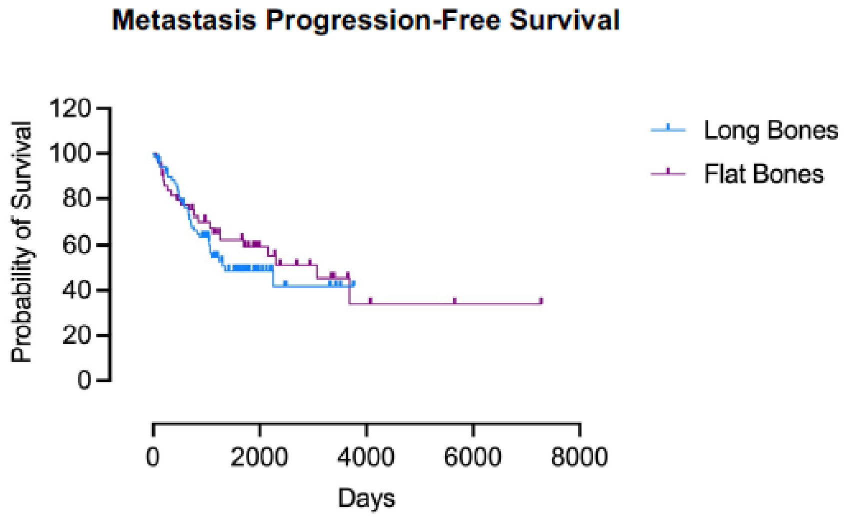

Figure S2. Kaplan-Meier survival curves for overall survival (**A**) and mestastasis progression-free survival (**B**) according to osteosarcoma location.

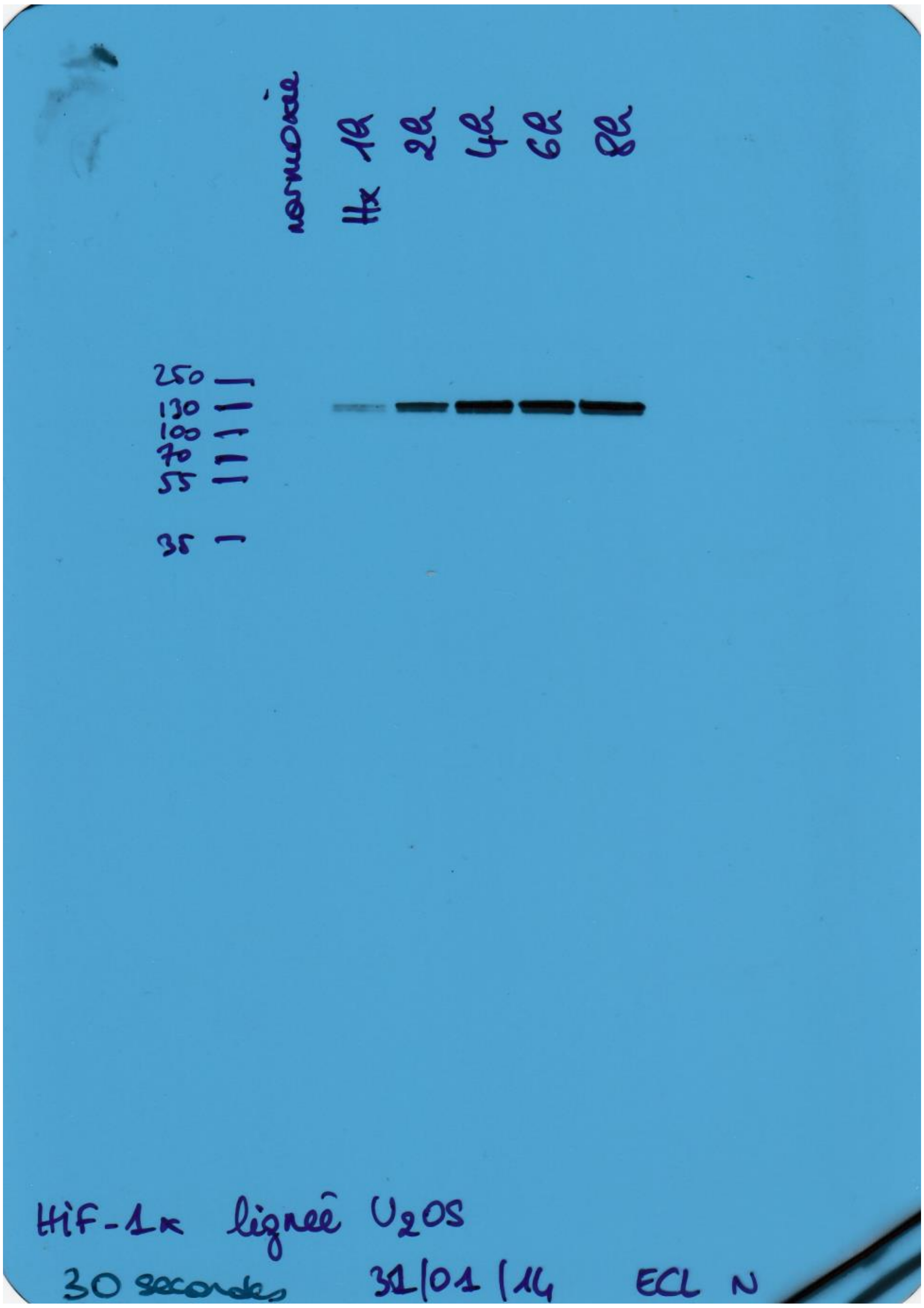

Figure S3. Original film (Figure 1A). U-2 OS / HIF1 staining.

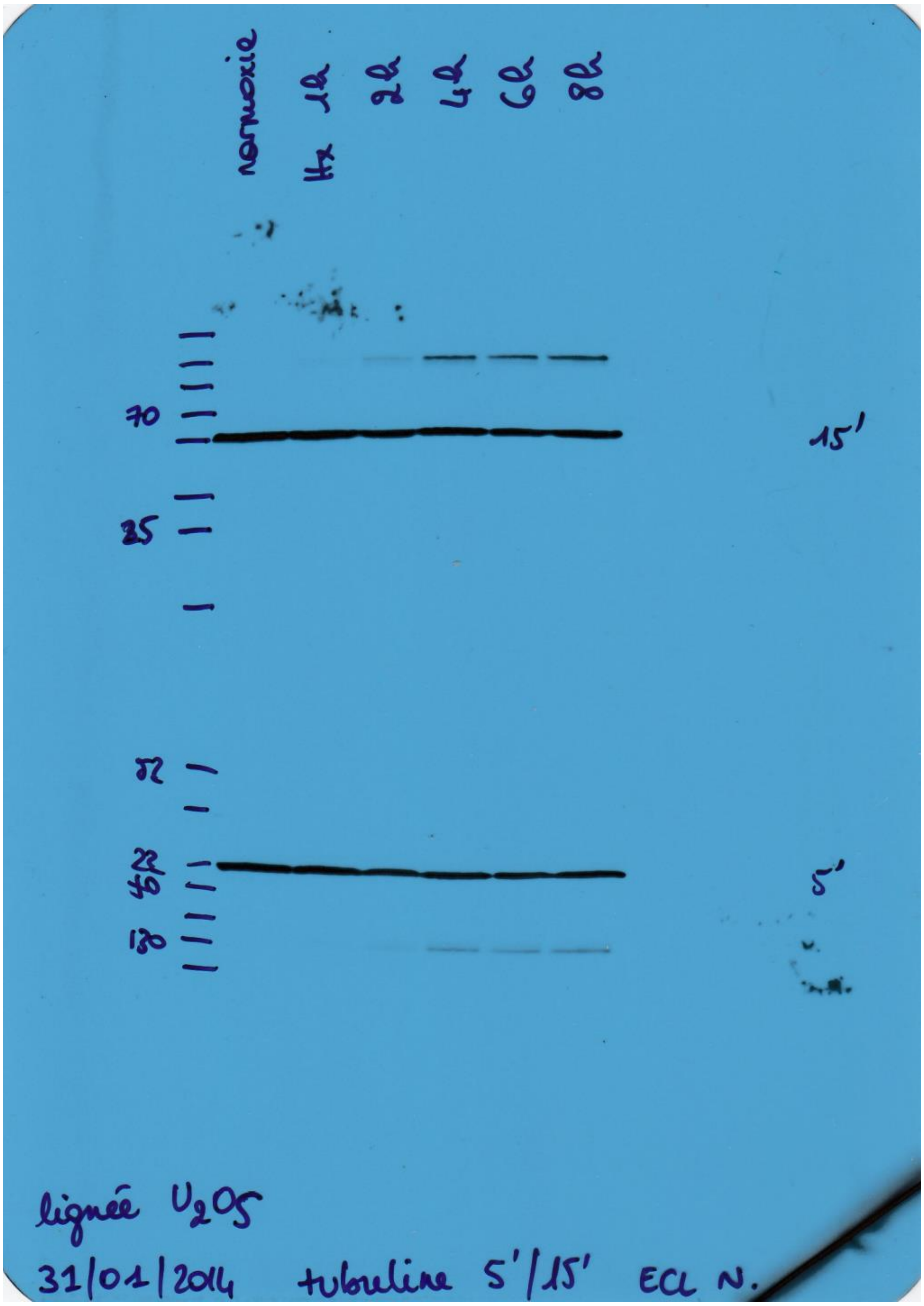

Figure S3. Original film (Figure 1A). U-2 OS / Tubulin staining.

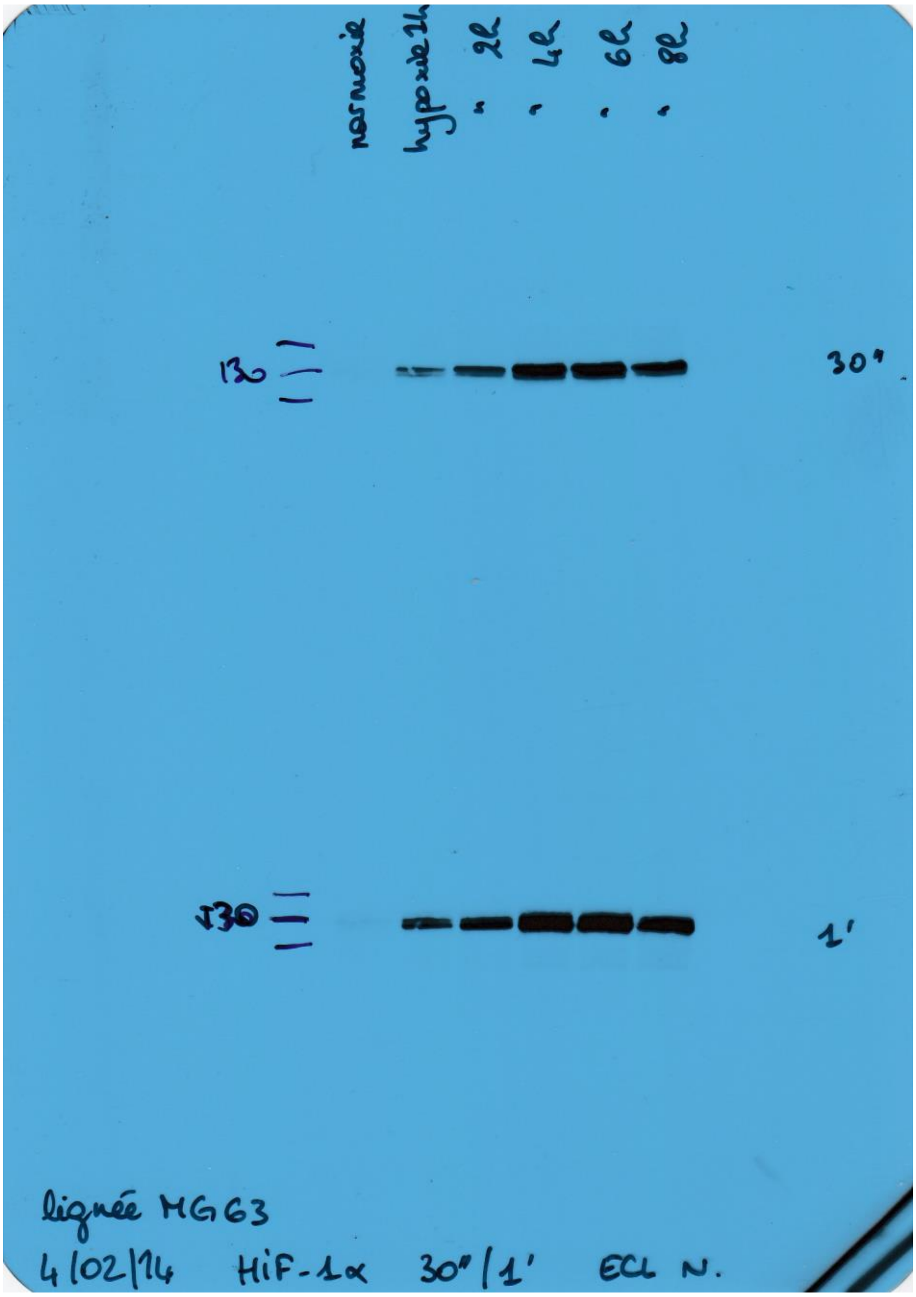

Figure S3. Original film (Figure 1A). MG-63 / HIF1 staining.

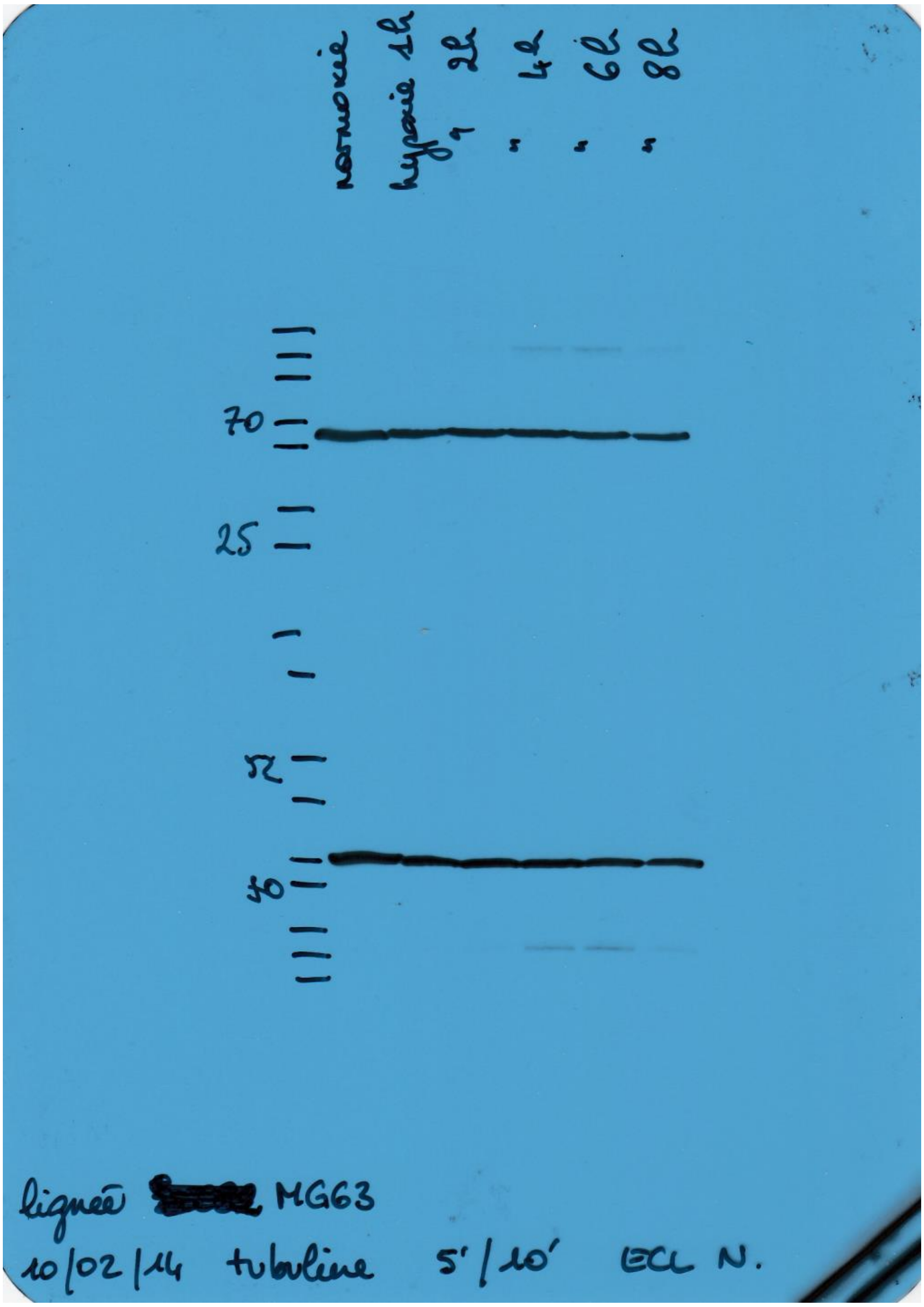

Figure S3. Original film (Figure 1A). MG-63 / Tubulin staining.

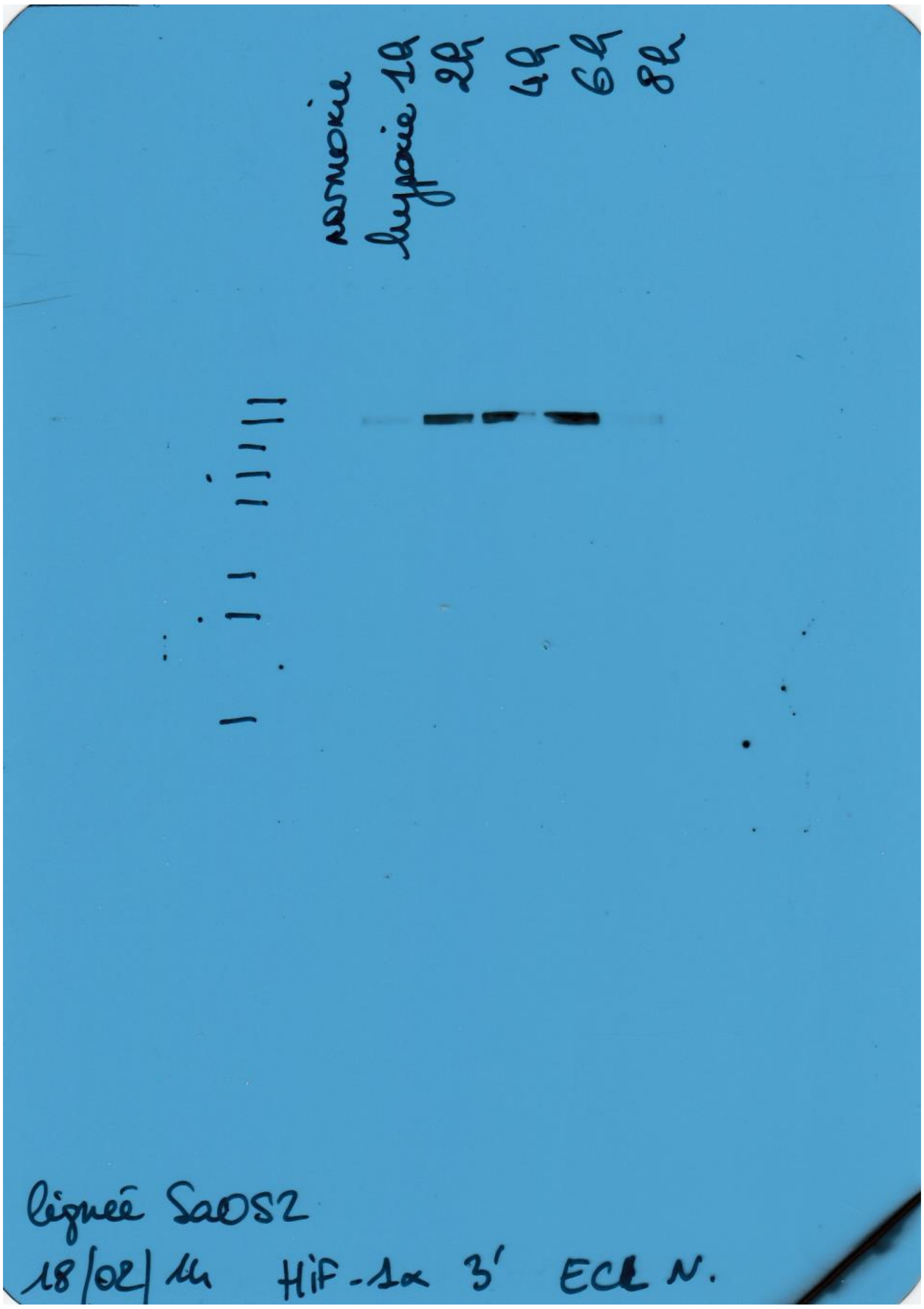

Figure S3. Original film (Figure 1A). SaOS-2 / HIF1 staining.

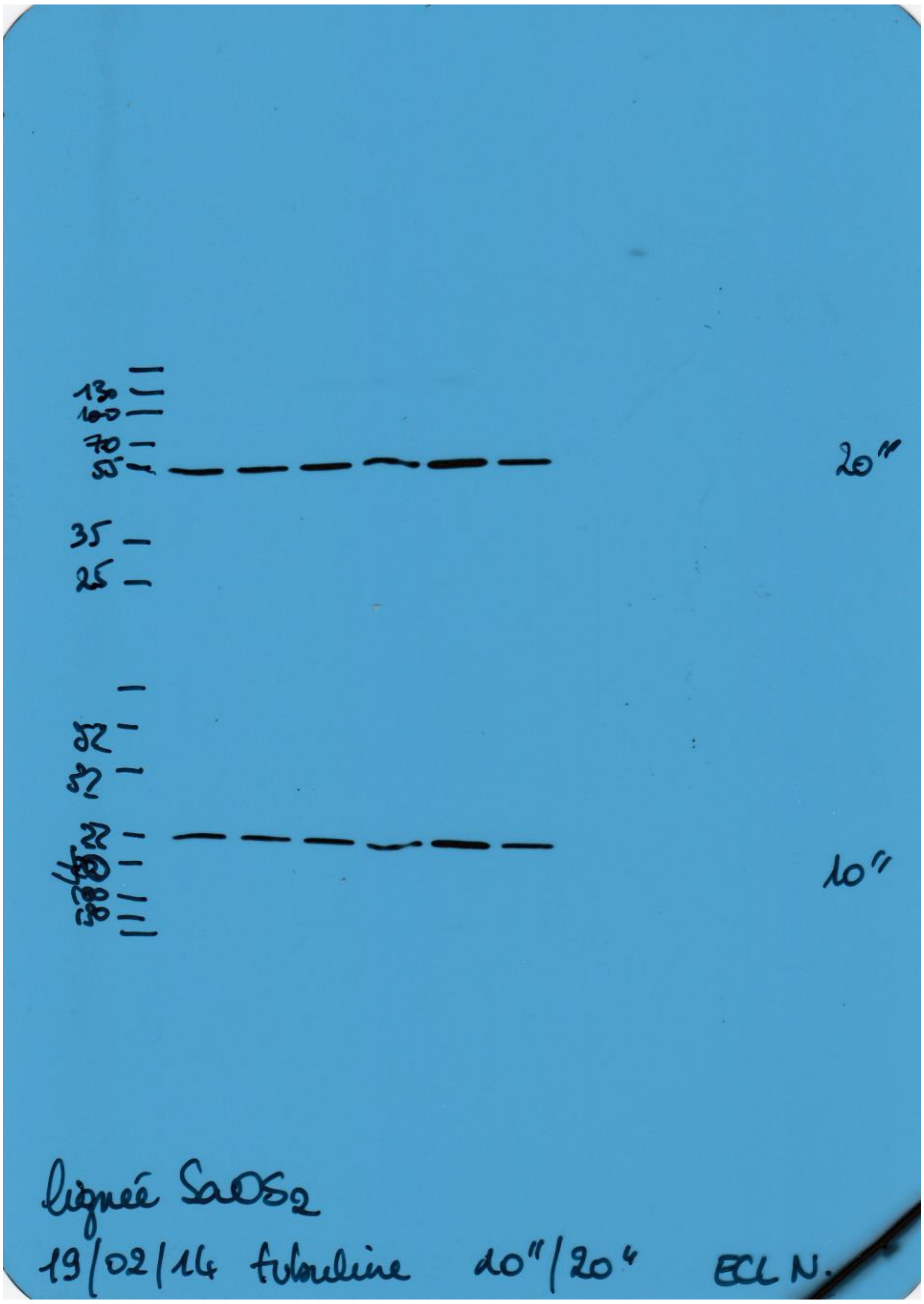

Figure S3. Original film (Figure 1A). SaOS-2 / Tubulin staining.

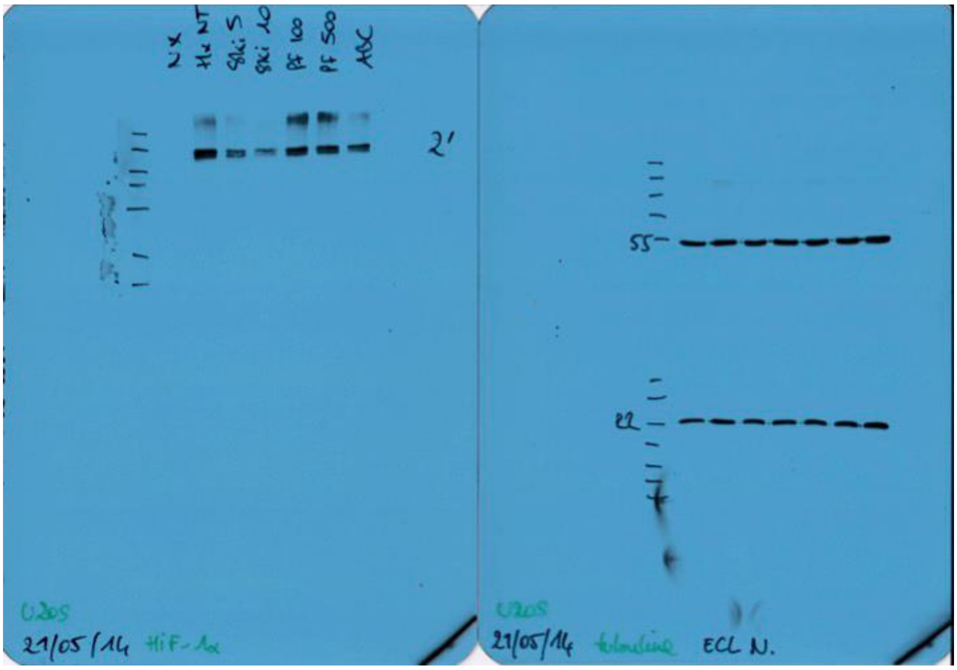

Figure S4. Original films. Figure 1D.

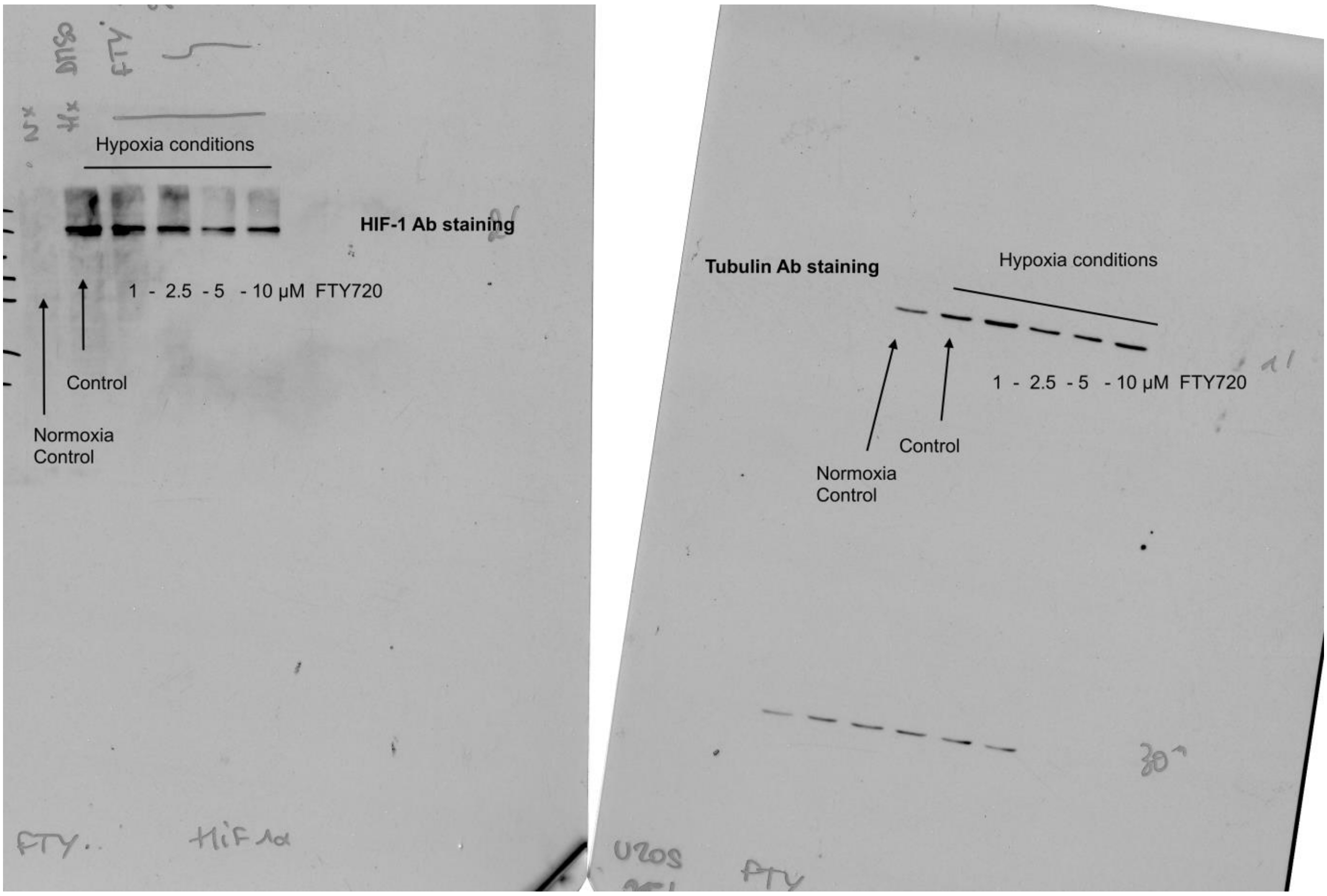

Figure S5. Original films (Figure 1E).

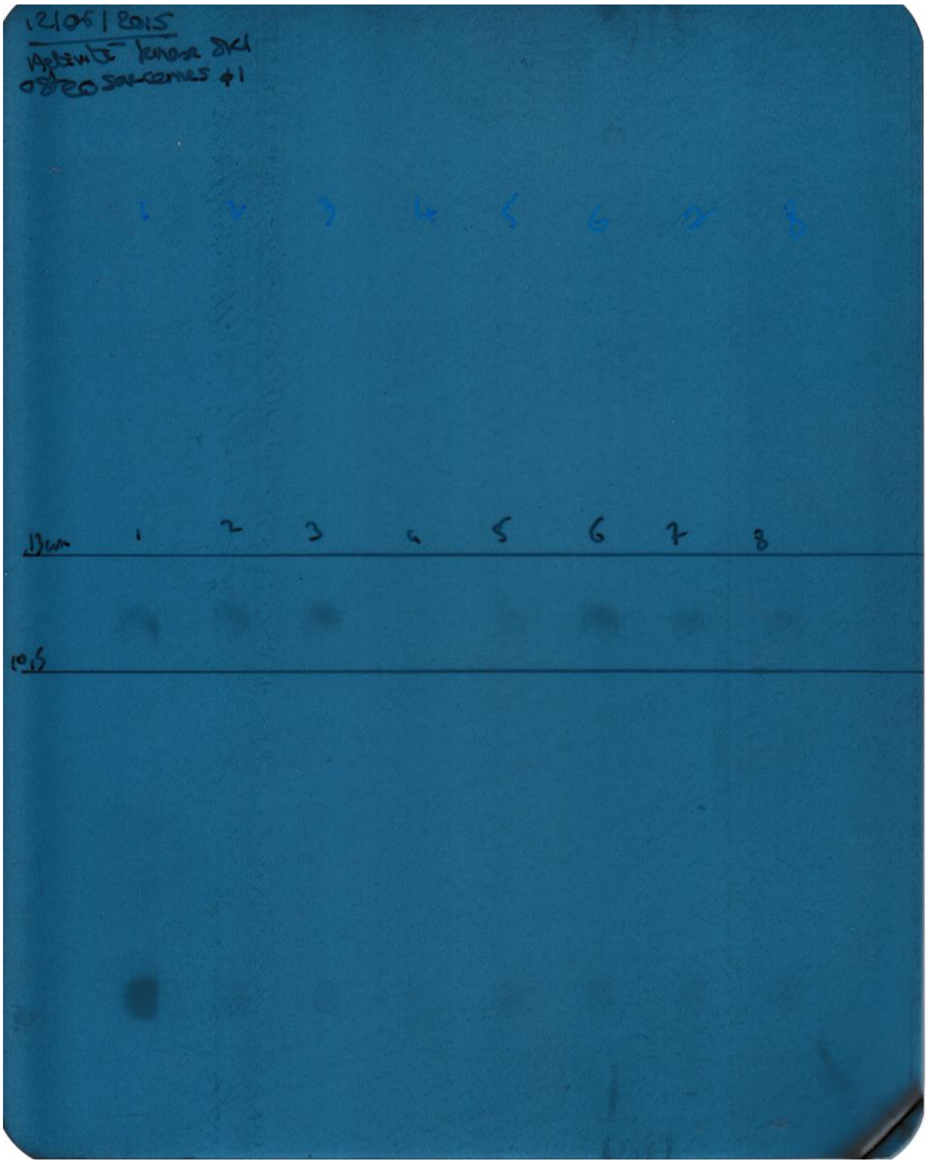
12/06/2015
1 2 3 4 5 6 7 8
1 2 3 4 5 6 7 8

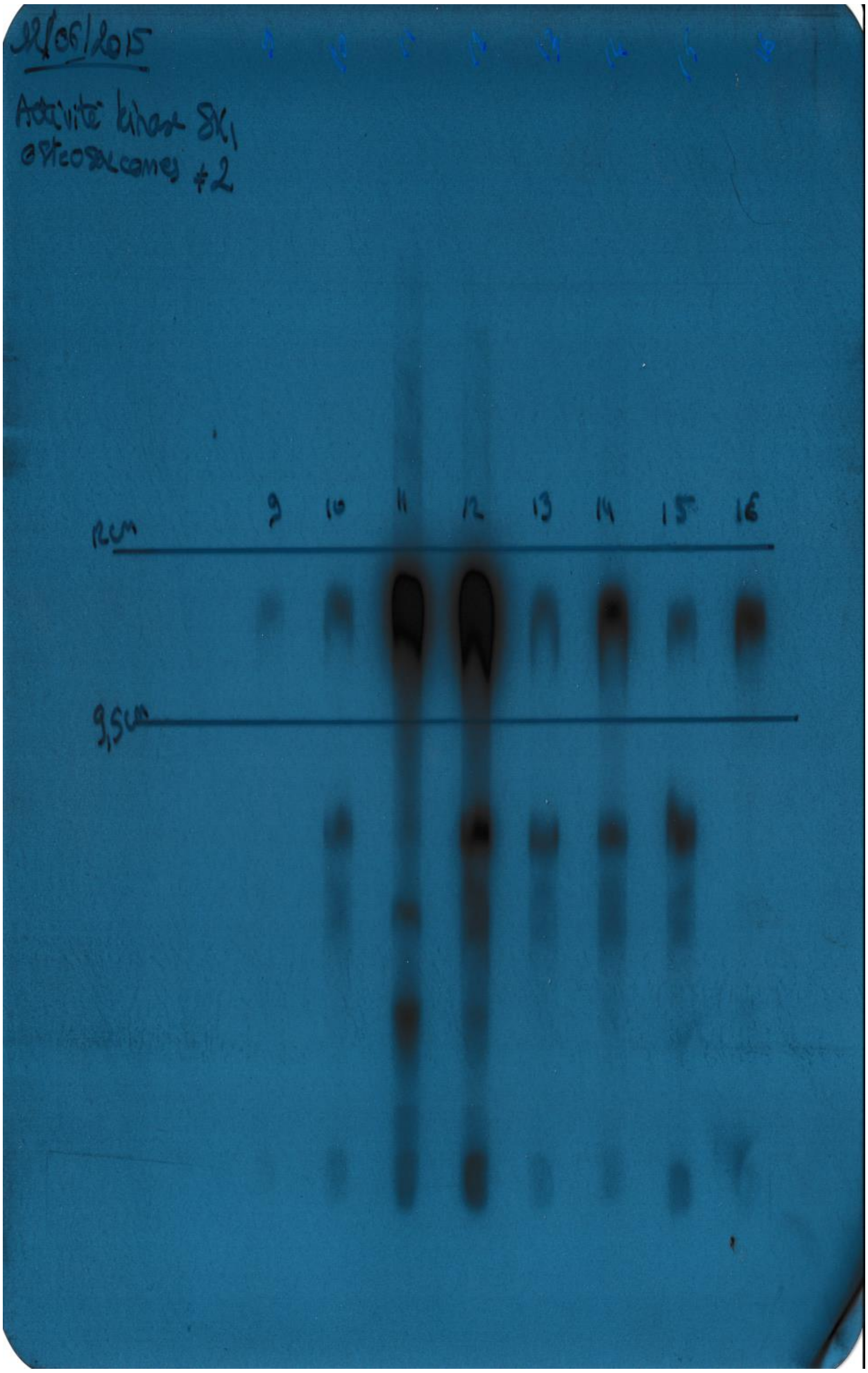
9
10
11
12
13
14
15
16
9,5cm

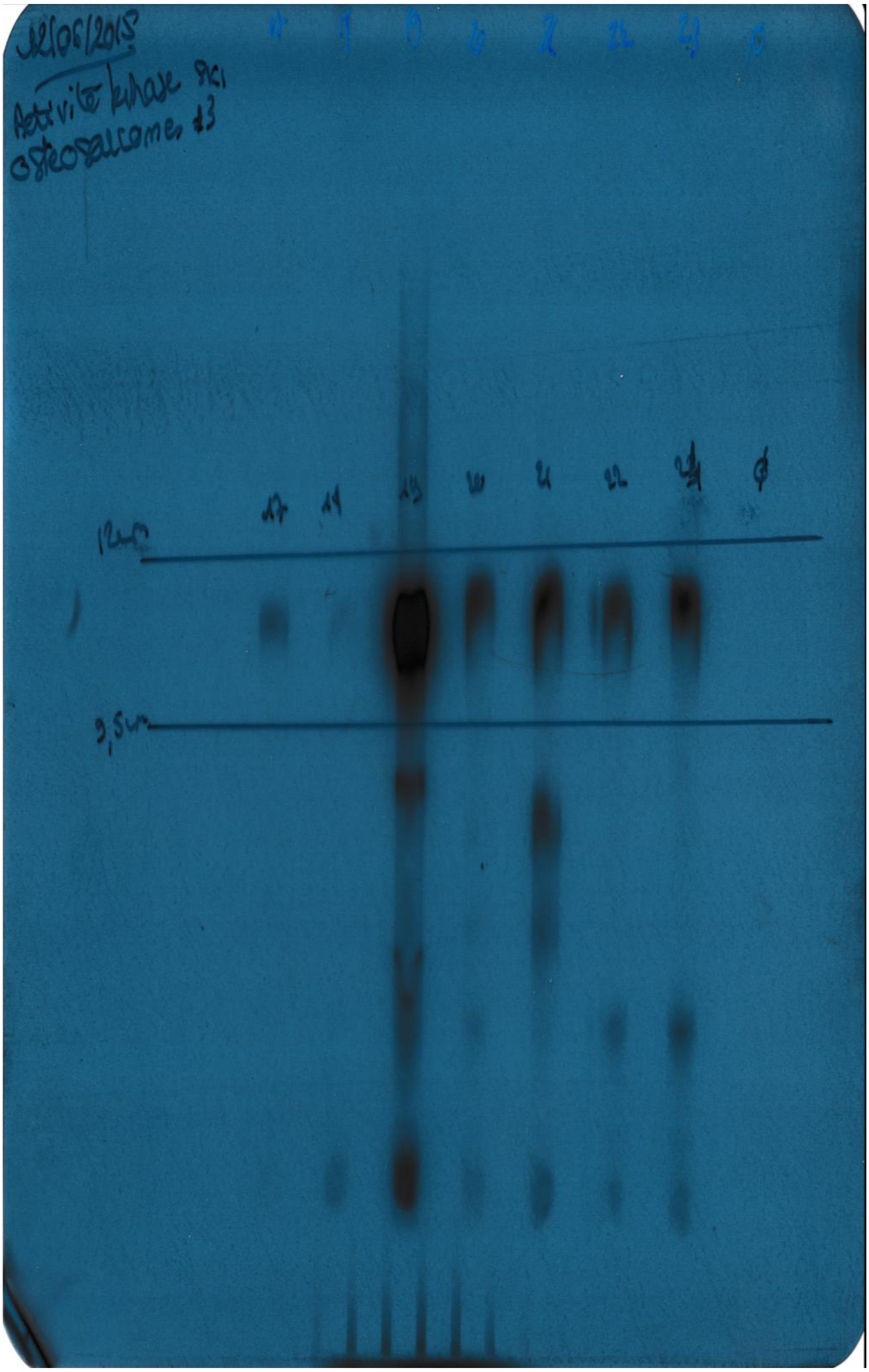

Figure S6. Original radiography obtained from Thin Layer Chromatography for experiment described in Figure 2A. Samples 1 to 10 are non-tumor samples (labelled as 1 to 10 in the figure); Samples 11 to 23 are tumor sample (labelled as 1 to 12 in the figure); Sample O = film background.

Table S1. Clinical parameters of good and poor responders to chemotherapy

**All OS**	**Good**		**Poor**		***p* value**
	***n* = 53**	**% 55.2**	***n* = 43**	**% 44.8**	
Age (Years)					
Median (Range)	16 (6–65)		31 (8–73)		<0.0001
≤25y	11	11.5	20	20.8	0.0004
>25y	42	43.7	23	24	
Gender					0.827
Male	34	35.4	27	28.1	
Female	19	19.8	15	15.6	
Unknown	0	0	1	1	
Metastatic vs Non Metastatic					0.5774
Localized	35	36.5	26	27.1	
Metastatic	18	18.8	17	17.7	
Progression					0.1911
0	33	34.4	21	21.9	
1	20	20.8	22	22.9	
Recurrence					0.2037
0	45	46.9	32	33.3	
1	8	8.3	11	11.5	
Death					0.0131
0	45	46.9	28	29.2	
1	8	8.3	15	15.6	
Long Bones					
	n = 39	% 66.1	*n* = 20	% 33.9	*p* value
Age (Years)					
Median (Range)	15 (6–47)		22 (8–60)		0.0137
≤25y	37	62.7	9	20.3	0.0049
>25y	2	3.4	11	13.6	
Gender					0.6501
Male	27	45.8	15	25.4	
Female	12	20.3	5	8.5	
Metastatic vs Non Metastatic					0.233
Localized	24	40.7	9	15.3	
Metastatic	15	25.4	11	18.6	
Progression					0.0838
0	23	39	7	11.9	
1	16	27.1	13	22	
Recurrence					0.2037
0	34	57.6	14	33.3	
1	5	8.5	6	11.5	
Death					0.027
0	33	55.9	13	22	
1	6	10.2	7	11.9	
Flat Bones					
	n = 14	% 37.8	*n* = 23	% 62.2	*p* value
Age (Years)					

Median (Range)	31 (6–65)		36 (15–73)		0.1924
≤25y	5	13.5	8	21.6	0.2187
>25y	9	24.3	15	40.5	
Gender					>0.9999
Male	7	18.9	12	32.4	
Female	7	18.9	10	27	
Unknown	0	0	1	2.7	
Metastatic vs Non Metastatic					0.7569
Localized	11	29.7	17	46	
Metastatic	3	8.1	6	16.2	
Progression					0.5274
0	10	27	14	37.8	
1	4	10.8	9	24.3	
Recurrence					0.9829
0	11	29.7	18	48.7	
1	3	8.1	5	13.5	
Death					0.1829
0	12	32.4	15	40.5	
1	2	5.4	3	8.1	

Table S2. Association between GLUT1, SphK1 and S1P1 biomarkers and OS, MPFS and good response to chemotherapy. Comparison between long bone and flat bone locations.

**Variable**	**Death (Overall Survival)**			**Death or Metastatic Progression (MPFS)**			**Good Response to Chemotherapy**		
	**Total**	**Long Bones**	**Flat Bones**	**Total**	**Long Bones**	**Flat Bones**	**Total**	**Long Bones**	**Flat Bones**
**GLUT1 (% of cells) ***									
n	98	54	44	98	54	44	70	44	26
HRa [IC 95%]	1.00 [0.90–1.11]	0.93 [0.81–1.06]	1.13 [0.95–1.35]	1.04 [0.95–1.14]	0.07 [0.87–1.08]	1.11 [0.94–1.32]	1.14 [0.98–1.34]	1.02 [0.84–1.25]	1.32 [0.97–1.81]
p	0.99	0.25	0.17	0.36	0.55	0.22	0.09	0.81	0.08
**SphK1 (% of cells) ***									
n	96	52	44	96	52	44	69	41	28
HRa [IC 95%]	1.0 [0.88–1.14]	1.97 [0.86–1.32]	0.95 [0.79–1.13]	1.03 [0.91–1.16]	1.04 [0.89–1.22]	0.98 [0.82–1.17]	1.02 [0.85–1.23]	1.12 [0.84–1.50]	0.86 [0.65–1.13]
p	0.99	0.55	0.55	0.66	0.6	0.79	0.82	0.44	0.27
**S1P$_1$ (% of cells) ***									
n	111	63	48	111	63	48	81	52	29
HRa [IC 95%]	1.13 [0.97–1.31]	1.31 [1.03–1.67]	0.96 [0.78–1.18]	1.11 [0.97–1.25]	1.22 [1.02–1.45]	0.93 [0.76–1.14]	0.86 [0.71–1.04]	0.71 [0.51–0.98]	1.04 [0.76–1.43]

p	0.11	0.03	0.67	0.12	0.03	0.5	0.86	0.01	0.81
**GLUT1 (IRS Score) ****									
n	98	54	44	98	54	44	70	44	26
HRa [IC 95%]	1.03 [0.94–1.11]	0.97 [0.87–1.09]	1.10 [0.96–1.26]	1.05 [0.98–1.12]	0.98 [0.90–1.08]	1.10 [0.96–1.25]	1.12 [0.99–1.27]	1.04 [0.89–1.23]	1.21 [0.96–1.52]
p	0.27	0.63	0.16	0.2	0.72	0.16	0.07	0.63	0.1
**SphK1 (IRS Score) ****									
n	96	52	44	96	52	44	69	41	28
HRa [IC 95%]	1.03 [0.91–1.17]	1.18 [0.99–1.40]	0.91 [0.74–1.11]	1.03 [0.92–1.15]	1.10 [0.94–1.30]	0.94 [0.78–1.14]	1.05 [0.87–1.26]	1.09 [0.81–1.47]	0.97 [0.74–1.26]
p	0.64	0.07	0.34	0.64	0.25	0.39	0.62	0.55	0.8
**$S1P_1$ (IRS Score) ****									
n	111	63	48	111	63	48	81	52	29
HRa [IC 95%]	1.07 [0.96–1.19]	1.09 [0.95–1.24]	1.01 [0.84–1.22]	1.07 [0.97–1.17]	1.09 [0.98–1.20]	1.00 [0.83–1.20]	0.86 [0.71–1.04]	0.76 [0.60–0.98]	1.09 [0.78–1.53]
p	0.24	0.22	0.9	0.18	0.12	0.99	0.11	0.03	0.62

* for a 10% increase of stained cells; ** for a 1 unit increase of IRS score.
